# Deep Kronecker LeNet for human motion classification with feature extraction

**DOI:** 10.1038/s41598-024-80195-7

**Published:** 2024-11-24

**Authors:** Thottempudi Pardhu, Vijay Kumar, Kalyan C. Durbhakula

**Affiliations:** 1 Department of Electronics and Communications Engineering, BVRIT HYDERABAD College of Engineering for Women, Hyderabad, 500090 India; 2 School of Electronics, Vellore Institute of Technology, Vellore, India; 3 Missouri Institute for Defense and Energy, University of Missouri-Kansas City, Kansas City, MO 64110 USA

**Keywords:** Human motion, Deep Kronecker network (DKN), LeNet, Spotted hyena optimizer (SHO), Grey wolf optimizer (GWO), Engineering, Mathematics and computing

## Abstract

Human motion classification is gaining more interest among researchers, and it is significant in various applications. Human motion classification and assessment play a significant role in health science and security. Technology-based human motion evaluation deploys motion sensors and infrared cameras for capturing essential portions of human motion and key facial elements. Nevertheless, the prime concern is providing effectual monitoring sensors amidst several stages with less privacy. To overcome this issue, we have developed a human motion categorization system called Deep Kronecker LeNet (DKLeNet), which uses a hybrid network.The system design of impulse radio Ultra-Wide Band (IR-UWB) through-wall radar (TWR) is devised, and the UWB radar acquires the signal. The acquired signal is passed through the gridding phase, and then the feature extraction unit is executed. A new module DKLeNet, which is tuned by Spotted Grey Wolf Optimizer (SGWO), wherein the layers of these networks are modified by applying the Fuzzy concept. In this model, the enhanced technique DKLeNet is unified by Deep Kronecker Network (DKN) and LeNet as well as the optimization modules SGWO is devised by Spotted Hyena Optimizer (SHO) and Grey Wolf Optimizer (GWO). The classified output of human motion is based on human walking, standing still, and empty. The analytic measures of DKLeNet_SGWO are Accuracy, True positive rate (TPR), True Negative rate (TNR), and Mean squared error (MSE) observed as 95.8%, 95.0%, 95.2%, and 38.5%, as well as the computational time observed less value in both training and testing data when compared to other modules with 4.099 min and 3.012 s.

## Introduction

Ultra-Wideband (UWB) technology, operating in the frequency range of 3.1–10.6 GHz, has been a significant breakthrough in radar sensing and wireless communication systems since its approval by the Federal Communications Commission (FCC) in 2002^1^. The key advantages of UWB systems, including high time resolution, low power consumption, excellent wall penetration, and simple hardware design, make them ideal for human motion classification in various scenarios like surveillance, healthcare, and rescue operations^2,3^. Impulse Radio Ultra-Wideband (IR-UWB), an early version of UWB, utilizes wideband pulses that allow for high-precision detection of human movements even through walls, thus enabling enhanced radar-based monitoring^4,5^.

IR-UWB radars work by transmitting short pulses and capturing reflected signals from objects and individuals, enabling precise human motion detection. These systems are used in modern smart homes, industrial automation, and healthcare environments where real-time detection of human activities is crucial^6,7^. Applications include smart lighting systems that adjust based on human presence, urban surveillance, and industrial safety where quick human detection helps avoid accidents^7,8^. Additionally, UWB radars’ ability to function in through-wall scenarios makes them invaluable in urban warfare, counter-terrorism, and search-and-rescue missions, where detecting human presence behind obstacles is critical^8,9^.

Despite these advancements, a significant gap exists in the literature concerning the computational efficiency and accuracy of current radar-based human motion classification models, especially in complex environments. Traditional machine learning approaches, such as support vector machines (SVMs) and time delay estimation, struggle to handle the high-dimensional and complex radar data^10^. While deep learning models like Convolutional Neural Networks (CNNs) and Long Short-Term Memory (LSTM) networks have shown improved classification accuracy, they still face challenges in dealing with cluttered environments, varying lighting conditions, and high computational demands^11,12^. Moreover, many existing models fail to generalize across different scenarios, limiting their use in real-time applications^11^.

The challenges in previous works primarily revolve around high computational costs and limited adaptability in dynamic environments. For instance, while models like TWR-MCAE and TwSense have shown some promise, they fall short in achieving real-time performance due to their computational overhead and lack of robustness in complex scenarios^10,13^. Furthermore, while optimization techniques such as Genetic Algorithms (GA) and Particle Swarm Optimization (PSO) have been employed, they often fail to fully optimize both feature extraction and classification processes^13,14^.

The novelty of this study lies in the development of a hybrid deep learning architecture, DKLeNet_SGWO, which combines Deep Kronecker Networks (DKN) and LeNet with the Spotted Grey Wolf Optimizer (SGWO). This architecture is designed to address the limitations of existing models by optimizing both feature extraction and classification processes, thereby achieving superior performance in terms of accuracy and computational efficiency. The integration of SGWO enables dynamic adjustment of model parameters, significantly reducing computational costs while maintaining high classification accuracy^15,16^. This unique approach ensures that the model is scalable and suitable for real-time applications like healthcare monitoring, urban surveillance, and search-and-rescue missions^17^.

In this study, the proposed DKLeNet_SGWO model classifies human movements into three categories: ambulation, immobility, and vacancy, using a robust combination of UWB radar signals, signal preprocessing, and advanced feature extraction methods. Additionally, the incorporation of fuzzy logic further enhances the model’s adaptability, ensuring it performs consistently across diverse environments^18^. This research provides a novel solution to the challenges posed by previous approaches and advances the field of radar-based human motion classification.

### Organization of the paper

The rest of the paper is structured as follows:

- Section 2 presents a detailed review of the issues and challenges in the past approaches to human motion classification using radar.
- Section 3 outlines the proposed system architecture, focusing on the DKLeNet_SGWO model and its components.
- Section 4 provides an in-depth analysis of the DKLeNet_SGWO model, including the feature extraction process and optimization using SGWO.
- Section 5discusses the performance evaluation of the proposed model, presenting experimental results and comparisons with state-of-the-art methods.
- Section 6 concludes the paper and suggests future directions for research in the area of radar-based human motion classification.

## Motivation

Human motion classification has been widely employed in many real applications, including within household environments, surveillance or search and rescue missions, or monitoring patients suffering from severe ailments. Especially in hospital scenarios, monitoring the body movements of the patients affected by severe diseases can be paramount. Hence, the researchers are encouraged to develop a new module in classifying human motion. The technique comprises inducing a study campaign and understanding the present models, identifying the difficulties of the present modules, and developing solutions for them.

### Literature survey

Ultra-Wideband (UWB) radar technology has become essential for detecting human motion, particularly in through-wall applications, due to its high-resolution capabilities and the ability to penetrate barriers. This innovation was made possible by the Federal Communications Commission (FCC), which, in 2002, established the regulations that allowed UWB technology to thrive^1^. Since then, UWB radar has evolved, finding applications in healthcare, security, and smart city infrastructures.

### Advancements in UWB for human detection

Kumar et al.^2^ explored advancements in UWB radar for human detection and monitoring. Their research highlighted how UWB radar outperforms traditional systems in cluttered environments, where the complexity of surroundings can impede accurate detection. They showcased UWB’s ability to provide precise and reliable data, making it a go-to technology for motion detection in complex settings.

Similarly, Singh et al.^4^ conducted a comprehensive review of through-wall human detection using UWB radar. The study pointed out the technology’s efficiency in security and military applications, where rapid detection through obstacles is critical. UWB’s penetration capability offers an advantage over traditional sensors, which are often hindered by solid materials like walls.

### UWB in healthcare and smart cities

In the healthcare domain, UWB radar has shown great promise in patient monitoring and smart homes. Zhang et al.^3^ reviewed UWB’s applications in healthcare and smart cities, emphasizing how this technology can be used for continuous health monitoring and activity recognition, particularly in elderly care and chronic illness management. The ability to detect even minute movements, such as breathing patterns, makes UWB radar ideal for non-invasive patient care.

Patel et al.^5^ explored UWB radar’s potential in human motion detection, focusing on its applications for detecting subtle movements such as respiration and heartbeat. This capability is crucial for healthcare monitoring, particularly for patients who require continuous observation without intrusive devices. Zhao et al.^6^ further examined UWB’s role in smart homes, particularly in monitoring human activities such as falls or abnormal movements, which are critical for ensuring the safety of elderly individuals living independently.

Lee et al.^7^ discussed how UWB radar can be integrated into smart cities for ambient sensing. The study focused on how this technology can monitor pedestrian movement, enhance urban safety, and manage traffic. By tracking human activities in real-time, UWB can contribute to building smarter and safer cities. Chen et al.^8^ also explored UWB’s application in urban warfare, where its ability to detect human motion through walls offers a strategic advantage in security and military operations.

### Machine learning and deep learning integration

The integration of machine learning with UWB radar has significantly improved detection accuracy and efficiency. Das et al.^10^ presented a survey on machine learning approaches for radar-based human motion detection, emphasizing the benefits of deep learning models like convolutional neural networks (CNNs) and recurrent neural networks (RNNs). These models can process large amounts of radar data quickly, making them ideal for real-time applications.

Smith et al.^11^ highlighted the role of deep learning in radar-based human motion classification, showing how CNNs can extract complex features from radar data, enhancing the precision of motion detection systems. Rahman et al.^12^ proposed novel deep learning architectures that significantly reduce computational complexity while maintaining high detection accuracy, making these systems suitable for real-time applications in surveillance and healthcare.

Park et al.^17^ developed deep learning models for human motion detection in complex environments. These models were designed for through-wall applications, where traditional sensors often struggle with interference and signal loss. The study highlighted the importance of machine learning in handling noisy environments and improving detection accuracy in challenging scenarios.

### Optimization techniques in UWB systems

Optimization techniques play a critical role in enhancing the performance of UWB radar systems. Wang et al.^15^ introduced the Spotted Grey Wolf Optimization (SGWO) algorithm, which improved feature selection and classification accuracy for radar-based motion detection. This technique optimized the radar system’s ability to detect motion in cluttered environments, making it more efficient.

Liu et al.^14^ applied Particle Swarm Optimization (PSO) to UWB radar systems, focusing on optimizing system parameters for better detection performance. Their research showed that PSO could significantly reduce the computational overhead of UWB systems while maintaining high accuracy, making it ideal for real-time applications where speed and efficiency are critical.

### Through-wall detection and rescue operations

Through-wall human motion detection has been one of the most impactful applications of UWB radar technology. Gao et al.^19^ applied genetic algorithms to optimize UWB-based human detection models, particularly in through-wall environments where signal interference is a major challenge. Their work improved the system’s ability to detect human motion even in complex environments with obstacles.

Kim et al.^9^ reviewed the use of UWB radar in search and rescue operations, where it is used to detect survivors in collapsed buildings or other disaster situations. The ability of UWB radar to penetrate rubble and detect motion has made it a valuable tool for rescue teams. Choi et al.^29^ built on this by developing a UWB-based system for real-time people counting, which can be applied in high-traffic areas or emergency response scenarios.

Wang et al.^21^ applied Conditional Generative Adversarial Networks (cGAN) to mitigate the effects of wiring and other environmental interferences on radar signals, significantly improving the accuracy of through-wall human motion detection. Sun et al.^22^ implemented a WiFi passive radar system for through-wall human sensing, demonstrating excellent performance in detecting motion but struggling with precise localization.

### Real-time and multi-task networks

Advanced UWB systems are now being designed to handle multiple tasks simultaneously. Lin et al.^24^ proposed a multi-task network that performs people counting, motion recognition, and localization using through-wall radar. This approach is particularly useful in real-time applications, where systems must handle multiple inputs and outputs efficiently.

Wang et al.^26^ developed Cycle-Consistent Generative Adversarial Networks (Cycle GAN) to de-noise radar signals and improve the accuracy of human motion detection. This approach is particularly effective in environments where signal interference is a persistent issue, such as urban areas or indoor environments with multiple reflective surfaces.

### Feature extraction and legacy works

Feature extraction techniques have been instrumental in improving the accuracy of UWB radar systems. Starck et al.^35^ introduced the Curvelet Transform, which has been widely used for radar signal processing due to its ability to capture directional features. Ahonen et al.^36^ developed the Local Phase Quantization (LPQ) method for extracting blur-invariant features, further enhancing the ability of UWB radar systems to detect subtle movements.

Pardhu et al.^49,50^, and^51^ conducted foundational research on through-wall radar imaging, focusing on detecting vital signs and human motion behind obstacles. Their work demonstrated the practical application of UWB radar in both healthcare and security sectors. Khan et al.^30^ developed algorithms for monitoring vital signs through UWB radar, providing a framework for modern non-invasive health monitoring systems.

Will et al.^31^ integrated Frequency-Modulated Continuous-Wave (FMCW) radar with UWB systems, enabling real-time tracking of human motion. This combination of technologies has expanded the use of radar for surveillance and monitoring purposes in both civilian and military contexts.

UWB radar has evolved to become a powerful tool for human motion detection, particularly in through-wall scenarios. The integration of machine learning and optimization techniques has further enhanced the performance of these systems, making them suitable for real-time applications in healthcare, smart cities, and rescue operations. However, challenges remain, especially in environments where signal interference is high. Future research will continue to focus on improving signal processing and feature extraction to overcome these limitations.

### Challenges

The challenges met in research on the classification of human motion are:

1. The model in^19^ simultaneously improved super features by reducing the low-rank characteristics of wall clutter but did not manage to balance computational cost and reliability.
2. Based on the factors above, in TwSense^20^, a user-friendly interface was meant to be explored for better application in Through-the-Wall (Tw) human motion recognition.
3. The solution of^21^ efficiently suppresses the impact of wiring upon the micro-Doppler signatures due to humans behind walls but at the cost of affecting motion detection and classification because of residual near-constant clutter components.
4. The method in^22^ demonstrated high sensitivity and expanded potential, but the convergence number of iterations varied according to different system parameters.
5. Human motion classification is complex because wall effects of attenuation, refraction, and multipath effects cause severe distortion to the echo signal, which would deform the echo signal beyond recognition, destroying classification accuracy or significantly increasing computation time.

## System model

The IR-UWB radar^34^ emits a sequence of electromagnetic waves at a specific frequency. These waves are generated by an antenna and used to detect objects through a channel. The object reflects the electromagnetic waves to the radar’s receiving antenna. The waves exhibit simplicity, which is advantageous for developing radar devices as it minimizes modulation and reduces complications associated with radar generation.

The electromagnetic wave is reflected by human movement using IR-UWB TWR with optimal range precision and captured by a receiver to produce an echo. The signal obtained from a human consists of various temporal delays caused by the scattering of the human body and its surrounding environment. The scattering coefficients of these objects varied, and the received signal from the UWB radar is depicted as follows:

1 $$\begin{aligned} \kappa (\nu )=\sum _{\mu =1}^{\nu } M_{\mu } f(\nu -\Phi _{\mu }) \quad \end{aligned}$$

In this context, $f(\nu )$ represents the transmitted signal, $\kappa (\nu )$ represents the received signal, $\nu$ represents the multipath reflection, $\Phi _{\mu }$ denotes the amplitude, and $\Phi _{\mu }$ represents the time delay.

The acquired echo signal is utilized to identify human motion at a specific moment, during which the signal’s progression is simulated. In this scenario, the radar signal does not diminish throughout transmission. The signal is represented as a Gaussian pulse signal, and the radar’s transceiver antenna is considered in an extreme state. The scattering center measures the movement of the chest and torso to determine whether a human is standing or walking. Therefore, the simulation receives a signal that is formulated by

2 $$\begin{aligned} \kappa (\nu )=f(\nu -\Phi _{jj}(\nu ))=f(\nu -(2 \times jj(\nu ))/qq) \quad \end{aligned}$$

where, $qq=3 \times 10^8 \text {m/s}$ that implies the propagation speed of waves, $\Phi _{jj}(\nu )$ indicates time delay as a path of human motion, $jj(\nu )$ depicts human path and function of time $\nu$. The simulation obtain signal matrix as $\textbf{B}[xx,\mu ]$ with rapid and slow time dimension is computed by,

3 $$\begin{aligned} \textbf{B}[xx,\mu ]= & \kappa [xx\textbf{A}_{uu},\nu \textbf{A}_{vv}]\nonumber \\= & -\textbf{E}_f \times \left( 2 \times [\nu \textbf{A}_{vv}-(2 \times jj(xx\textbf{A}_{uu}))/qq]\right) / \alpha ^2 \nonumber \\ & \times e^{-(\nu \textbf{A}_{vv}-(2 \times jj(xx\textbf{A}_{uu}))/qq)/\alpha ^2} \quad \end{aligned}$$

Here, “sampling points” refer to *xx*, while “$\textbf{A}_{uu}$” represents the slow time direction. “$\nu$” explains the concept of sampling points, and “$\textbf{A}_{vv}$” defines the fast time direction. “$M_f$” shows the amplitude of the transmit signal, and “$\alpha$” represents the pulse width factor.

### Dataset and preprocessing

The IR-UWB Through-Wall Radar Human Motion Status Dataset, used to train and evaluate the proposed DKLeNet_SGWO model, contains radar signal data that captures various human activities through walls. The dataset consists of a substantial collection of samples, recorded under different conditions to capture three distinct human motion classes: walking, standing still, and empty room. In total, the dataset comprises 10,000 radar signal samples, ensuring a diverse and robust set of data for training and testing.

### Feature extraction

Feature extraction plays a critical role in radar signal analysis for human motion classification. A variety of advanced techniques were applied to extract informative features from the radar signals. These features are vital in enhancing the model’s ability to differentiate between various human motion patterns. Specifically, 150 characteristics were extracted from each radar signal sample, categorized into the following key characteristic types.

1. **Curvelet Transform**: This transform captures multi-scale geometrical structures, particularly useful for detecting curves and edges, which are essential for understanding the complex motion patterns inherent in radar signals.
2. **Local Phase Quantization (LPQ) with Discrete Wavelet Transform (DWT)**: LPQ helps extract blur-invariant local phase information, while DWT decomposes the radar signals into different frequency bands. These features are particularly effective at capturing both spatial and frequency information, improving noise resilience and overall classification accuracy.
3. **Speeded-Up Robust Features (SURF)**: SURF detects key local features that are invariant to scale and rotation. This helps in identifying motion patterns such as walking or subtle movements in standing still.
4. **Statistical Features**: To further characterize the radar signals, statistical metrics such as mean, variance, standard deviation, and entropy were extracted. These features quantify the distribution and complexity of the radar signals, providing essential information about the signal’s underlying structure.

### Training and testing setup

The dataset was split into 90% training data and 10% testing data to ensure sufficient training for the deep learning model while maintaining a separate test set for unbiased performance evaluation. Specifically:

1. **Training data size:** 9000 radar signal samples
2. **Testing data size:** 1000 radar signal samples

The training set was utilized to optimize the model’s parameters through multiple iterations, ensuring the model’s capacity to generalize across different motion patterns. The testing set was exclusively reserved for performance evaluation, providing a reliable measure of the model’s ability to generalize to unseen data.

### Training parameters and hyperparameter tuning

The proposed **DKLeNet_SGWO** model was trained with a set of carefully selected parameters to optimize its performance in human motion classification. The following key training parameters were used:

- **Learning Rate**: A constant learning rate of **0.001** was applied throughout the training process. This value was chosen based on preliminary experiments to ensure steady convergence without causing the model to overshoot minima during optimization.
- **Batch Size**: A batch size of **64** was utilized to balance between computation efficiency and model performance. This batch size allowed for sufficient gradient updates per epoch without overwhelming the GPU resources.
- **Number of Epochs**: The model was trained for a total of **100 epochs**. This value was determined empirically by monitoring the validation loss and accuracy, ensuring that the model had enough epochs to converge without overfitting the training data.
- **Optimizer**: The **Spotted Grey Wolf Optimizer (SGWO)** was employed as the primary optimization algorithm. SGWO dynamically adjusted the weights and biases during training. This optimizer was chosen for its ability to balance exploration (searching the global space) and exploitation (refining known optimal regions), which proved crucial for enhancing the model’s accuracy and achieving efficient convergence.

#### Hyperparameter definition

The hyperparameters of the convolutional neural network architecture in **DKLeNet_SGWO** were fine-tuned using a combination of grid search and empirical testing. The following hyperparameters were defined:

- **Number of Convolutional Layers**: The network contains **four convolutional layers**, each responsible for progressively extracting higher-level features from the radar signals.
- **Kernel Sizes**: The kernel sizes varied between **3x3** and **5x5**, depending on the layer. Smaller kernel sizes were used in earlier layers to capture fine-grained features, while larger kernel sizes in later layers were useful for broader feature extraction.
- **Activation Function**: The **ReLU (Rectified Linear Unit)** activation function was applied after each convolutional layer to introduce non-linearity and allow the model to learn complex patterns in the radar signals.
- **Pooling Layers**: Max-pooling layers followed each convolutional layer, reducing the spatial dimensions of the feature maps and preventing overfitting by down-sampling the feature representations.

The **SGWO** algorithm, combining elements of the Spotted Hyena Optimizer (SHO) and the Grey Wolf Optimizer (GWO), was specifically chosen for its efficiency in optimizing both the network’s weights and bias parameters. This hybrid optimization technique allowed the model to converge more rapidly and reduced the Mean Squared Error (MSE) during testing, enhancing both accuracy and computational efficiency.

## Proposed DKLeNet_SGWO for human motion classification

**Figure 1:**
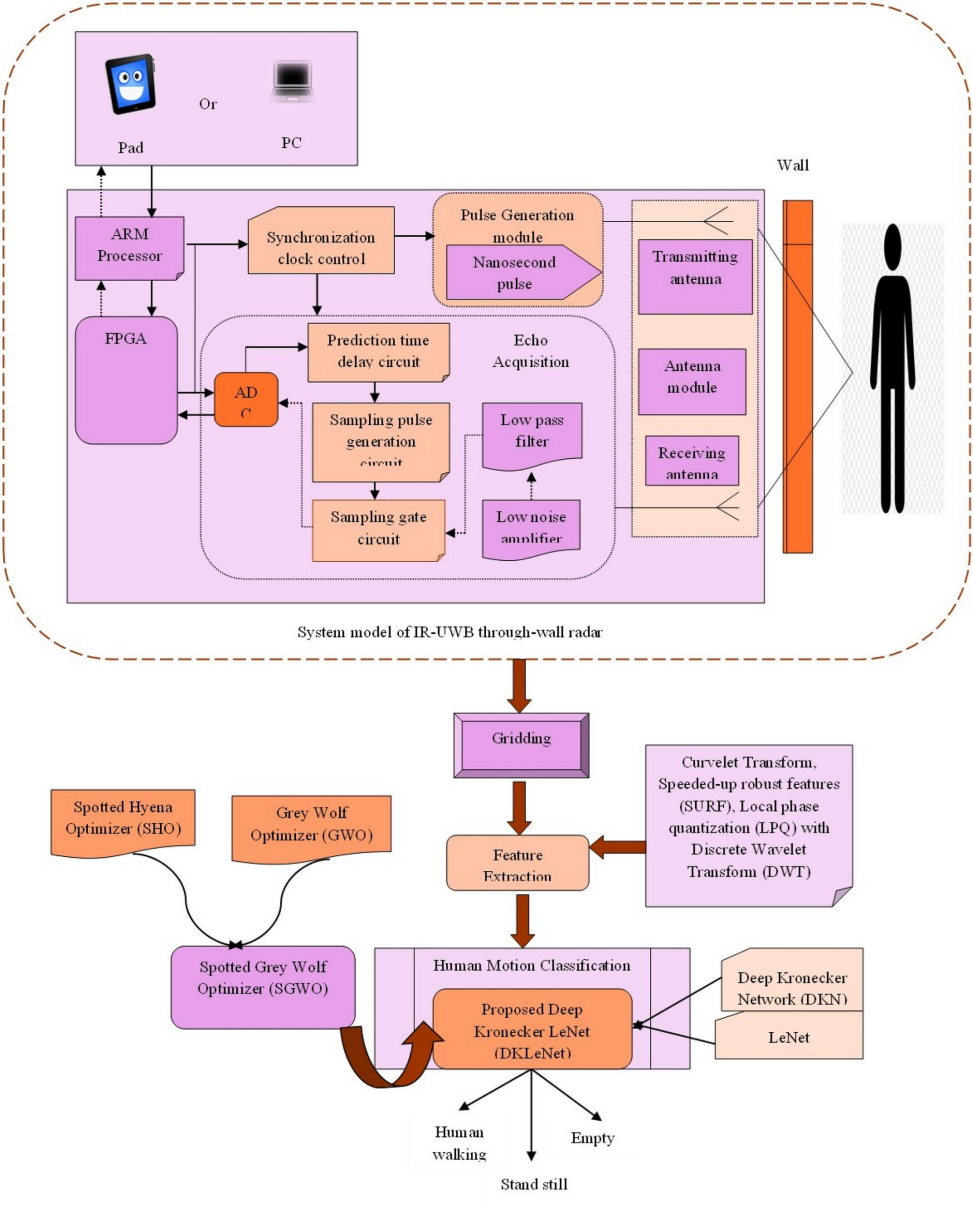
Demonstration of DKLeNet_SGWO for human motion classification.

**Figure 2:**
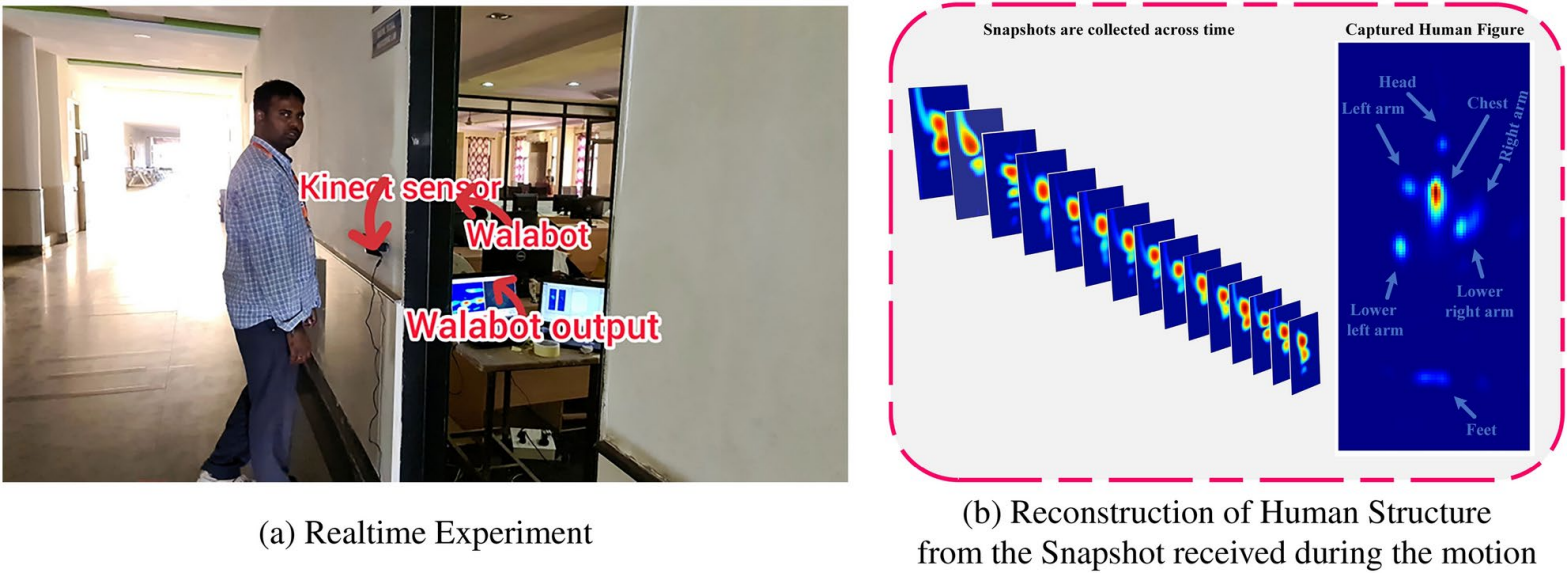
Illustration of experimental setup and data reconstruction in through-the-wall radar detection.

The main objective of this research is to provide a novel framework for categorizing human movement by utilizing the DKLeNet_SGWO module. DKLeNet is the combination of DKN^39^ and LeNet^40^. The initial design of the system module for IR-UWB TWR is developed. Here, the system includes the Advanced RISC Machine (ARM), Field-Programmable Gate Arrays (FPGAs), Analogue to Digital Converter (ADC), Pulse Generation Module, as well as transmission and receiver antennas. Subsequently, the UWB radar detects the signal and transmits it to the gridding unit to generate a series of grids and perform the feature extraction procedure. The retrieved features include Curvelet Transform^35^, Speeded-up robust features (SURF)^38^, Local phase quantization (LPQ)^36^ with Discrete Wavelet Transform (DWT)^37^, and statistical features^44^ such as mean, variance, standard deviation, and entropy. Human motion categorization uses DKLeNet_SGWO, which modifies the layers by incorporating fuzzy concepts. Here, the SGWO algorithm is used with the SHO algorithm^42^ and the GWO algorithm^43^. Furthermore, human movement is categorized as walking, stationary, and empty. Figure 1 is a clear and illustrative presentation of DKLeNet_SGWO for human motion classification. Figure 2 explains the real time experimental setup and the Reflections from the different Body of Human Behind the wall.

### Signal acquisition and gridding

Assuming *n* signals in a traditional database for classifying human motion, this is calculated as follows:

4 $$\begin{aligned} H = \{H_1, H_2, \ldots , H_m, \ldots , H_n\} \quad \end{aligned}$$

The input signal is considered to be $H_m$, and the total number of signals is denoted by $H_n$.

The gridding is carried out to achieve a set of grids for each signal. This involves applying grids to make the task easier by implementing simple steps. This process simplifies data generation for exploration and navigation, enabling data prediction. The radar signal is first analyzed as a radar Doppler spectral image, and then the image is further processed. In this scenario, an unstable object is detected, and its location is analyzed by capturing its position at different intervals.

#### Gridding to separate the distance samples

Input signal $H_m$ is utilized to examine the target’s location using gridding progress, which is subsequently classified into additional grids that enable the detection of the identical location. The set of grids denoted as $G_m$ is represented by a total count of grids *q*. It can be portrayed as

5 $$\begin{aligned} G_m = \{G_1, G_2, \ldots , G_p, \ldots , G_q\} \quad \end{aligned}$$

The gridding process is accomplished by utilizing a time and distance image plot. The features are subsequently extracted utilizing the grid $G_p$.

### Extraction of features

The process is utilized to identify the most compact and distinct patterns, thereby enhancing the classifier’s effectiveness. Additionally, it is employed to extract features from the actual signal, aiming to achieve accurate classification. In this phase, ’grid $G_p$’ serves as the input, which is provided and then processed to obtain superior feature vectors. The features extracted through this process are listed as follows.

#### The curvelet transform

The 2D series of ridgelet transform^35^ in $\mathbb {R}_r$ is represented with smooth univariate function $\hat{\psi }: \mathbb {R}_r \rightarrow \mathbb {R}_r$ also with sufficient decay and assuring the admissibility condition that handles when it indicates $\hat{\psi }$ has vanishing average $\int _{\mathbb {R}} \hat{\psi }(t) dt = 0$ and also assumed a particular normalization of $\hat{\psi }$, such that $\int _{\mathbb {R}} |\hat{\psi }(\lambda )|^2 \lambda ^{-2} d\lambda = 1$.

6 $$\begin{aligned} \int _{\mathbb {R}} |\hat{\psi }(\lambda )|^2/|\lambda | d\lambda < \infty \quad \end{aligned}$$

Every $b_b \in \mathbb {R}_r$ for each $a_a > 0$ as well as $\theta \in [0,2\pi ]$, then bivariate ridgelet $\hat{\psi }_{a,b,\theta }: \mathbb {R}_r^2 \rightarrow \mathbb {R}_r$ is given as,

7 $$\begin{aligned} \hat{\psi }_{a,b,\theta }(x) = a^{-\frac{1}{2}} \cdot \hat{\psi }\left( \frac{(x_1 \cos \theta + x_2 \sin \theta - b)}{a}\right) \quad \end{aligned}$$

A ridgelet is invariable around the lines $x_1 \cos \theta + x_2 \sin \theta = const$, where transverse to these ridges as wavelet. By providing an integrable bivariate function *f*(*x*), the ridgelet coefficients are defined as,

8 $$\begin{aligned} R_rf(a,b,\theta ) = \int _{\mathbb {R}^2} \overline{\hat{\psi }_{a,b,\theta }(x)} f(x) dx \quad \end{aligned}$$

The original regeneration representation is formulated as,

9 $$\begin{aligned} f(x) = \int _{\mathbb {R}}\int _{\mathbb {R}}\int _{0}^{2\pi } R_rf(a,b,\theta ) \hat{\psi }_{a,b,\theta }(x) \frac{da}{a^3} db d\theta /4\pi \quad \end{aligned}$$

The above mentioned expression validated for functions that are both integrable and square integrable. The valuation is generated as wavelet evaluation in radon field. Recollect radon transform of object *f* is the group of line integrals represented as $(\theta ,t) \in [(0,2\pi ) \times \mathbb {R}]$ that is illustrated as,

10 $$\begin{aligned} R_r f(\theta ,t) = \int _{\mathbb {R}^2} f(x_1,x_2) \varepsilon (x_1 \cos \theta + x_2 \sin \theta - t) dx_1 dx_2 \quad \end{aligned}$$

Here, Dirac distribution implies $\varepsilon$ and then ridgelet transform is accurately the application of 1D wavelet transform to radon transform fragments wherein angular variable implies $\theta$ is constant and *t* is differing. This feature is performed with the input grid of $G_p$. By applying this feature to the input grid, it is categorized onto four kinds of bands like low-low band, high-high band, high-low filter and low-high band. By applying this, low-low band signal is achieved, which is denoted as $F_1$.

#### LPQ with DWT

This feature applies the input $G_p$ to DWT, which is then classified into four bands that are already mentioned in the above feature. Here high-low band is neglected since it has large amount of noise. The LPQ feature is applied to the other three bands, which is concatenated and resulted as final outcome.

**Discrete Wavelet Transform (DWT)**

Here, the wavelet is a rapidly vanishing oscillating function progressed in the time domain and frequency. The signal is decomposed into scaled as well as translated versions $\rho _{\psi ,\lambda }(l)$ of a single function $\rho (l)$ in a series of wavelet evaluations computed by,

11 $$\begin{aligned} \rho _{\psi ,\lambda }(l) = \frac{1}{\sqrt{|\psi |}} \rho \left( \frac{l-\lambda }{\psi }\right) \quad \end{aligned}$$

Here, scale and translation factors represent $\psi ,\lambda$ with $\psi ,\lambda \in \mathbb {R}$ and $\psi \ne 0$. DWT is attained by altering the factors $\psi ,\lambda$. By substituting the value of $\psi =2^o$ and $\lambda =k 2^{o}$ into the above equation,

12 $$\begin{aligned} \rho _{o,k}(l) = 2^{-\frac{o}{2}} \rho \left( 2^{-o} l - k\right) \quad \end{aligned}$$

Thus, DWT^37^ is computed by,

13 $$\begin{aligned} m_{o,k} = \int _{-\infty }^{+\infty } s(l) 2^{-\frac{o}{2}} \rho ^*(2^{-o} l - k) dl = \langle s(l), \rho _{o,k}(l) \rangle \quad \end{aligned}$$

Here, wavelet coefficients signify $m_{o,k}$ at level *o* and location *k*.

**Local Phase Quantization (LPQ)**

LPQ^36^ is based on the property of blur invariance using Fourier phase spectrum and it deploys local phase information extracted by 2D discrete Fourier transform (DFT). In LPQ, it is evaluated in local neighborhoods at every grid position $\chi$ of signal $f(\chi )$. These local spectra are determined by short-term Fourier transform that is illustrated as,

14 $$\begin{aligned} f(\Omega ,\chi ) = \sum _{\Upsilon \in \mathcal {N}_\chi } f(\chi - \Upsilon ) e^{-j2\pi \Omega ^T \Upsilon } \quad \end{aligned}$$

The transform in Eq. (14) is effectively analyzed for entire positions for rows and columns using 1D convolutions. The unit information in Fourier coefficients is stored by real and imaginary parts of every element in $f(\chi )$, which is performed by a simple scalar quantizer that is indicated as,

15 $$\begin{aligned} \partial _l(\chi ) = {\left\{ \begin{array}{ll} 1, & \text {if } \pi _l(\chi ) \ge 0 \\ 0, & \text {otherwise} \end{array}\right. } \quad \end{aligned}$$

Here, *l*-th element of vector $\textbf{H}_\chi = [\text {Re}\{f_\chi \}, \text {Im}\{f_\chi \}]$ implies $\pi _l(\chi )$. The resulting eight binary coefficients $\partial _l(\chi )$ are illustrated by integer values among (0, 255) employing binary coding, which is formulated by,

16 $$\begin{aligned} f_{\text {LPQ}}(\chi ) = \sum _{l=1}^{8} \partial _l(\chi ) 2^{(l-1)} \quad \end{aligned}$$

Thus, the label signal of $f_{\text {LPQ}}$ is attained wherein the values are blur invariant LPQ. The extracted feature is illustrated as $F_2$.

#### Speeded-up robust features (SURF)

This feature^38^ extracts local robust features by applying a Hessian detector and distribution descriptor. Here, the input is $G_p$. In order to identify the interest points, this applies a Hessian-based blob detector. The determination of the Hessian detector enumerates the enlargement of response and local alteration around the area is illustrated by,

17 $$\begin{aligned} \textbf{M}(k,\delta ) = \begin{bmatrix} R_{ii}(k,\delta ) & R_{ki}(k,\delta ) \\ LL_{ki}(k,\delta ) & R_{ii}(k,\delta ) \end{bmatrix} \quad \end{aligned}$$

The distribution descriptor deliberates with the dimension of 20 that divides 4$\times$4 sub-fields and it is decrypted by wavelet response values that is enumerated as,

18 $$\begin{aligned} \textbf{T}_t = \left\{ \sum {dk}\sum {|dk|}\sum {di}\sum {|di|} \right\} \quad \end{aligned}$$

The extracted feature of SURF implies $F_3$.

#### Statistical features

Here, the statistical features^44^ are applied to the extracted features $F_1$, $F_2$, $F_3$ for attaining the final feature vectors with the aid of mean, variance, standard deviation, and entropy, which are described below.

i) Mean This refers to the total data concentration of the distribution that is computed as,
  19 $$\begin{aligned} w_1 = \sum _{t=0}^{V-1} t \cdot \mathbb {Z}(t) \quad \end{aligned}$$
  Here, the total number of grey levels implies *V*, $\mathbb {Z}(t)$ indicates probability, and $w_1$ refers to the mean.
ii) Variance This indicates the deviation value relevant to the mean value, which is calculated by,
  20 $$\begin{aligned} w_2 = \sum _{t=0}^{V-1} (t - w_1)^2 \cdot \mathbb {Z}(t) \quad \end{aligned}$$
  Here, variance enumerates $w_2$.
iii) Standard deviation It is the evaluation of the total dissimilarity or diffusion of an enormous amount of values that is exploited as,
  21 $$\begin{aligned} w_3 = \sqrt{\sum _{t=0}^{V-1} (t - w_1)^2 \cdot \mathbb {Z}(t)} \quad \end{aligned}$$
  Here, standard deviation indicates $w_3$.
iv) Entropy This is applied for measuring the complexity and the information comprised in the signal that is determined by,
  22 $$\begin{aligned} w_4 = \sum _{t=0}^{V-1} \mathbb {Z}(t) \cdot \log _2[\mathbb {Z}(t)] \quad \end{aligned}$$
  Here, entropy denotes $w_4$.

Thus, by applying the statistical features over extracted feature, the feature vector is illustrated as,

23 $$\begin{aligned} F^1&= \{w_1^1, w_2^1, w_3^1, w_4^1\} \quad \end{aligned}$$

24 $$\begin{aligned} F^2&= \{w_1^2, w_2^2, w_3^2, w_4^2\} \quad \end{aligned}$$

25 $$\begin{aligned} F^3&= \{w_1^3, w_2^3, w_3^3, w_4^3\} \quad \end{aligned}$$

Therefore, the feature vector is demonstrated by,

26 $$\begin{aligned} F_p = \{F^1, F^2, F^3\} \quad \end{aligned}$$

### Human motion classification using DKLeNet

**Figure 3:**
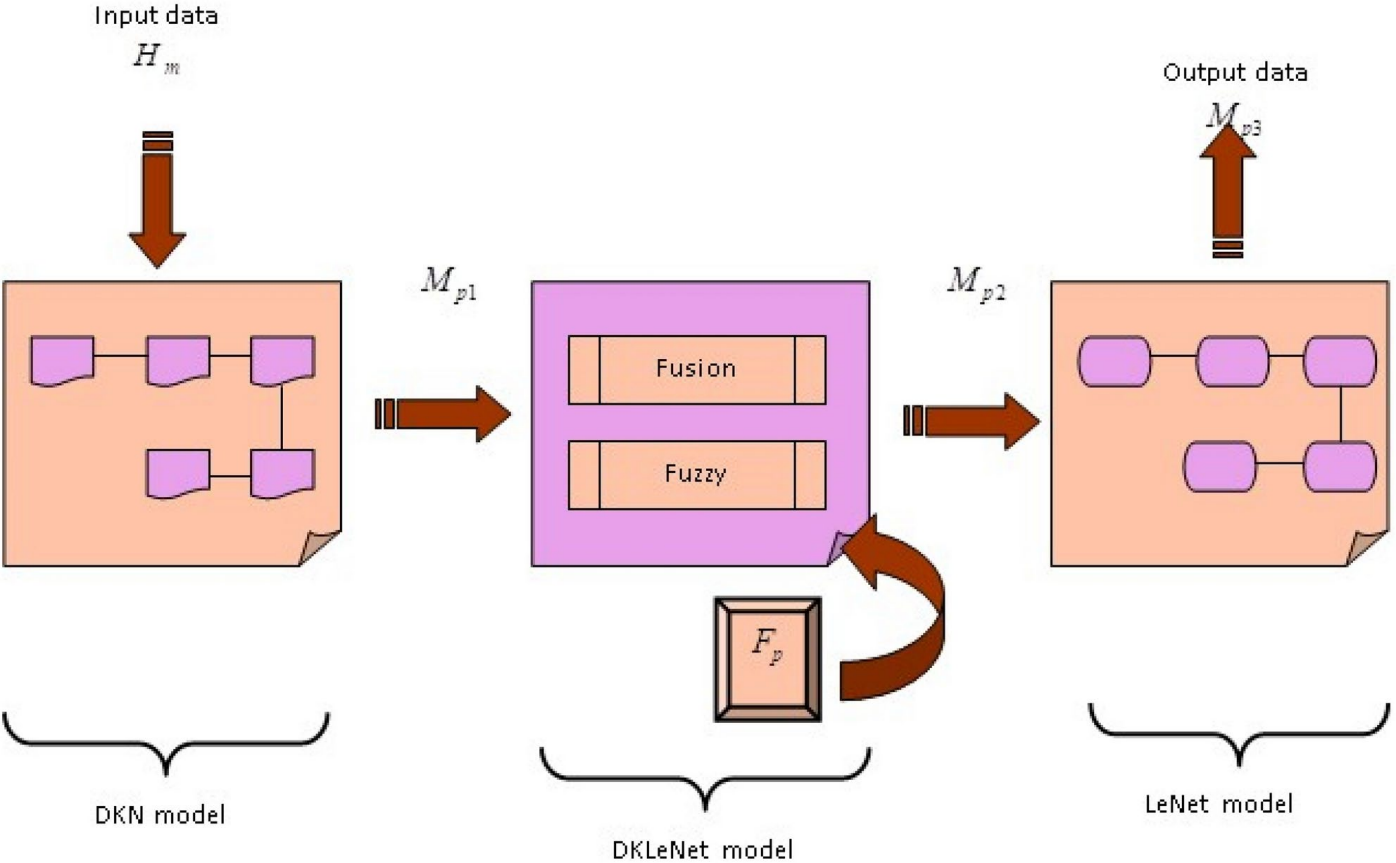
Illustration demonstration of DKLeNet.

Human motion classification is essential for supporting various sectors, particularly healthcare. In this sector, there is a critical need for the capability to monitor patients’ activities, mainly to provide services that include rapid response when necessary. In the proposed system, the developed technique comprises three models: DKN, DKLeNet, and LeNet. In the DKN model, the input signal $H_m$ is processed and yields an output denoted as $M_{p1}$. This output is then passed to the next layer, DKLeNet, resulting in $M_{p2}$. Furthermore, this intermediate output is fed into the final module, culminating into the final model output, $M_{p3}$. The structure of DKLeNet is illustrated in Fig. 3.

#### Structure of DKN

DKN^39^ is introduced based on the Kronecker product and completely requires a latent piecewise smooth property of coefficients. DKN is also provided for locating the signals, which is significant to the resultant and aids the evaluation of the module, and this network operates for high order tensor and matrix signals.

In order to initiate this progress, here the number of samples along with matrix-indicated images $W_e \in \mathbb {R}^{b \times c}$ as well as scalar responses $a_e, e = 1, \ldots , \epsilon$. Considering $a_e$ deploys a generalized linear technique, which is represented by,

27 $$\begin{aligned} M_{p1} = \tau (a_e) \exp \left\{ a_e \langle W_e, J \rangle - \varpi (\langle W_e, J \rangle )\right\} \quad \end{aligned}$$

Here, the target unknown coefficient matrix represents $J \in \mathbb {R}^{b \times c}$, a specific known univariate function implies $\tau (\cdot )$ and $\varpi (\cdot )$.

Based on the aforesaid expression, the analysis of the image is focused and neglects other variables like age and gender, which is included again in the module simples if it is significant. The specific known link function $h(\cdot )$, which is formulated by,

28 $$\begin{aligned} h(\mathcal {A}(a_e)) = \langle W_e, J \rangle \quad \end{aligned}$$

With the aid of DKN, the coefficient *J* is introduced with rank Kronecker factor decomposition with $U (\ge 2)$ is illustrated by,

29 $$\begin{aligned} J = \sum _{s=1}^S D_U^s \otimes D_{U-1}^s \otimes \cdots \otimes D_1^s \quad \end{aligned}$$

Here, unknown matrices depict $D_u^s \in \mathbb {R}^{b_u \times c_u}, u=1, \ldots , U; s=1, \ldots , S$ which is also implied as Kronecker parameters. The dimension of $D_u^s$ are not considered to be a known value. Nevertheless, since the property of Kronecker product, they specifically required to assure $b = \prod _{u=1}^U b_u$ and $c = \prod _{u=1}^U c_u$. For a simple representation, it is enumerated as for any matrices $D_{u'}, \ldots , D_{u''}$ with $u' \ge u''$,

30 $$\begin{aligned} D_{u'} \otimes D_{u'-1} \otimes \cdots \otimes D_{u''} = \otimes _{\perp (u=u')}^{(u'')} D_\kappa \quad \end{aligned}$$

Thus, Eq. (29) is recomputed as,

31 $$\begin{aligned} J = \sum _{s=1}^S \otimes _{\perp (u=U)}^{(1)} D_u^s \quad \end{aligned}$$

**Figure 4:**
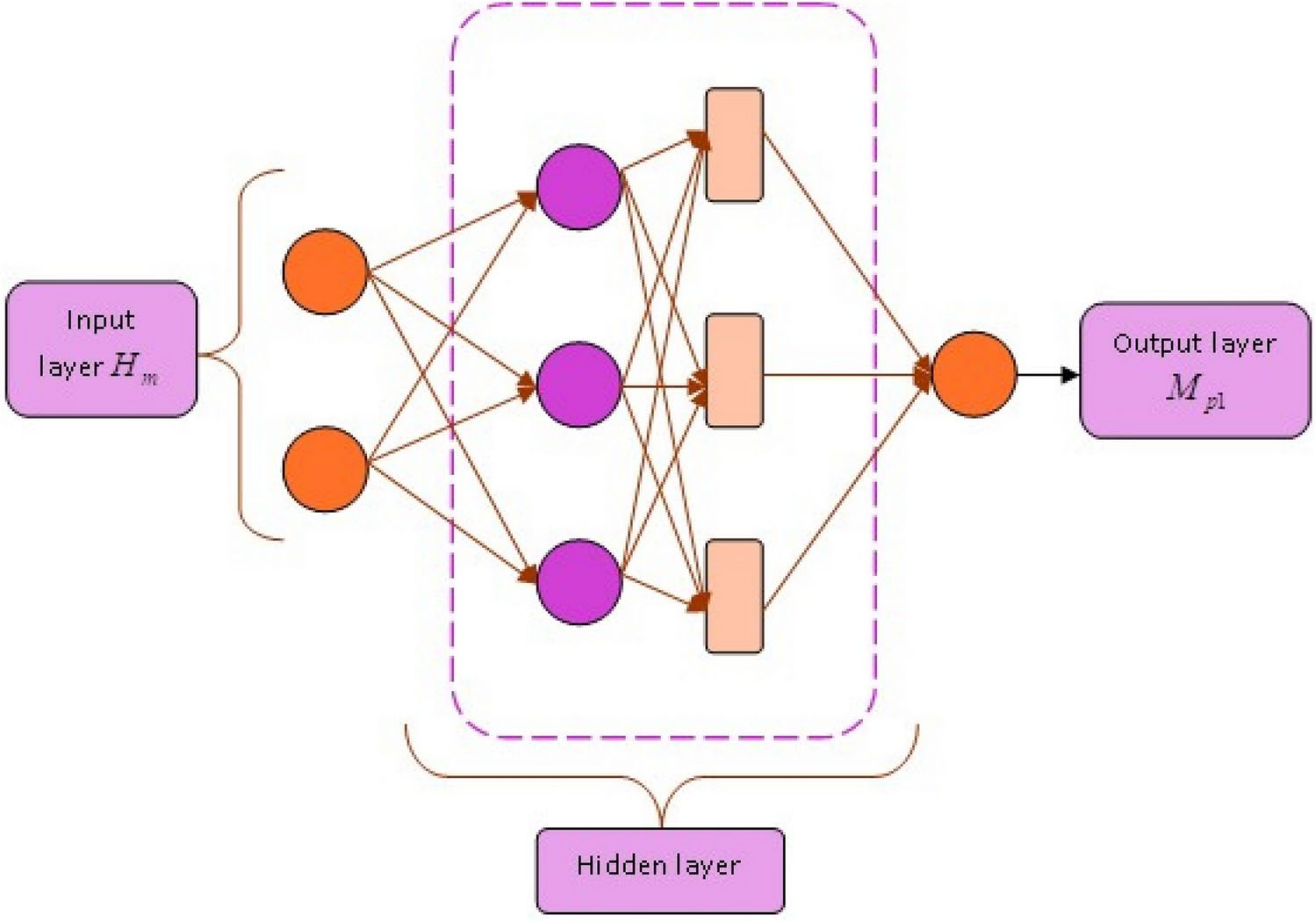
Structure of DKN.

This signifies decomposition with rank $S=2$ and parameter value $U=3$ for sparse matrix with signal turning a circle. Generally, the decomposition in Eq. (29) is capable for analyzing random matrix with adequate large rank *S*. The above expressions are referred to as DKN since it is similar to the fully convolutional network (FCN). Especially, *S* and *U* may be represented by the width and depth of DKN. The DKN resultant indicates $M_{p1}$ and its modeled diagrammatic view is designed in Fig. 4.

#### Structure of DKLeNet

In this layer, fusion and regression units are carried out with the input of $M_{p1}$ and extracted feature $F_p$. The fusion is applied for integrating two structures and fuzzy is to authenticate the equivalence amongst targeted as well as classified output. The layers are modified by performing fuzzy concept^41^. **Classification problem** Consider the classification issues in 2D pattern space $[0,1] \times [0,1]$ for enhancing the graphical representation with *j* patterns $z_l=(z_{l1},z_{l2}), l=1,2,\dots ,j$ are provided as training data from *E* number of classes. By applying Taylor concept,

32 $$\begin{aligned} z(r+1)= & z(r)+\frac{z'(r)}{1!} \quad \end{aligned}$$

33 $$\begin{aligned} z'(r)= & \frac{z(r)-z(r+1)}{v} \quad \end{aligned}$$

Let us assume $v=1$, substitute $z'(r)$ in Eq. (32),

34 $$\begin{aligned} z(r+1)= & z(r)+\frac{z(r)-z(r+1)}{1!} \quad \end{aligned}$$

35 $$\begin{aligned} z(r+1)= & 2z(r)-z(r+1) \quad \end{aligned}$$

36 $$\begin{aligned} z_l=2 \times F_p - M_{m1} \quad \end{aligned}$$

Here, the feature vector expounds $F_p$ and resultant of former layer depicts $M_{m1}$.

**Fuzzy partition** By categorizing every axis of pattern space into $N (N \ge 2)$ along with the subsets of $\{Q_1^N, Q_2^N, \dots , Q_N^N\}$. Here, triangular membership function *s* is applied that is denoted as $Q_g^N$.

37 $$\begin{aligned} \theta _g^N (z) = \max \{1 - \frac{|z - \alpha _g^N|}{\beta ^N}, 0\}, \quad g=1,2,\dots ,N (N \ge 2) \quad \end{aligned}$$

Here, membership function of $Q_g^N$ implies $\theta _g^N (z)$.

38 $$\begin{aligned} \alpha _g^N = \frac{(g-1)}{(N-1)}, \quad g=1,2,\dots ,N \quad \end{aligned}$$

39 $$\begin{aligned} \beta ^N = \frac{1}{(N-1)} \quad \end{aligned}$$

In such case, $N=1$, then membership function of $Q_1^1$ is computed as,

40 $$\begin{aligned} \theta _1^1 (z) = {\left\{ \begin{array}{ll} 1 & \text {if } 0 \le z \le 1 \\ 0 & \text {otherwise} \end{array}\right. } \quad \end{aligned}$$

Here, $Q_1^1$ is in the rage of [0, 1].

**Rule generation** When $z_{l1}$ is $Q_g^K$ and $z_{l2}$ is $Q_g^L$, $(z_{l1},z_{l2})$ is based on class $I_{go}^{KL}$ with $cf=cf_{go}^{KL}, g=1,2,\dots ,K; K=1,2,\dots ,K_{\text {max}}; o=1,2,\dots ,L; L=1,2,\dots ,L_{\text {max}}$. Here, $I_{go}^{KL}$ indicates *E* classes as well as certainty of fuzzy rule imply $cf_{go}^{KL}$, every rule of both terms is examined based on beneath progresses.

Examination of fuzzy rules

1. Determine $\lambda _{ct}$ for $t=1,2,...,E$, which is formulated as,
  41 $$\begin{aligned} \lambda _{ct} = \sum _{z_l \in ct} \theta _g^K(z_{l1}) \times \theta _o^L(z_{l2}) \end{aligned}$$
2. Identify class *x*(*cx*) such that it is illustrated by,
  42 $$\begin{aligned} \lambda _{cx} = \max \{\lambda _{c1},\lambda _{c2},\ldots ,\lambda _{ce}\} \end{aligned}$$
  When more than two classes consider maximal values in the aforesaid expression, the consequent of fuzzy rule based on its subspace is not examined appropriately. When single class considers maximal value $\lambda _{cx}$, $I_{go}^{KL}$, it is examined as class *x*(*cx*) in $\lambda _{cx}$.
3. When single class considers maximal value in Eq. (42), then $I_{go}^{KL}$ is computed by,
  43 $$\begin{aligned} I_{go}^{KL} = \frac{(\lambda _{cx} - \lambda )}{\sum _{t=1}^{E}\lambda _{ct}} \end{aligned}$$
  Here,
  44 $$\begin{aligned} \lambda = \frac{\sum _{t=1}^{E}\lambda _{ct}}{(E-1)} \end{aligned}$$
  Here, $cf_{go}^{(KL)}$ is computed by class *x*(*cx*) that has maximal sum of $\theta _g^K(z_{l1}) \times \theta _o^L(z_{l2})$ amongst E classes in Eq. (42). The regulations of fuzzy in subsequent portion are fake regulations, which have no impact on fuzzy inference to classify new patterns. When there is no pattern in fuzzy subspace, fake regulation is created by this progress at fuzzy subspace. Consider a set of entire fuzzy rules by $O_{all}$, which is determined by,
  45 $$\begin{aligned} O_{all} = \{P_{go}^{KL}; g=1,2,\ldots ,K; K=1,2,\ldots ,K_{\text {max}}\} \end{aligned}$$
  Moreover, the set of fuzzy rules in every fuzzy partition by $O^{KL}$
  46 $$\begin{aligned} O^{KL} = \{P_{go}^{KL}; g=1,2,\ldots ,K; K=1,2,\ldots ,K_{\text {max}}\} \end{aligned}$$
  Here, $O^{KL}$ implies fuzzy rules in partition by $K \times L$ fuzzy grid, the regulation set $O_{\text {all}}$ of fuzzy rules are determined by,
  47 $$\begin{aligned} O_{\text {all}} = \bigcup _{K=1}^{K_{\text {max}}}\bigcup _{L=1}^{L_{\text {max}}}O^{KL} \end{aligned}$$
  The concern based on rule selection is for selecting essential fuzzy rules and for neglecting unrelated fuzzy rules from $O_{\text {all}}$.

**New pattern classification** Consider a subset *O* of $O_{\text {all}}$ is provided for creating fuzzy classification system. By utilizing these rules in *O*, a new pattern $z_l=(z_{l1},z_{l2})$ is categorized as follows:

1. Determine $\gamma _{ct}$ for $t=1,2,\ldots ,E$ by
  48 $$\begin{aligned} \gamma _{ct}&= \max \{\theta _g^K(z_{l1}) \times \theta _o^L(z_{l2}) \times cf_{go}^{KL} : \nonumber \\&\quad c_{go}^{KL} = \text {class} \wedge P_{go}^{KL} \in O\} \end{aligned}$$
2. Identify class *x*(*cx*), then
  49 $$\begin{aligned} M_{m2} = \gamma _{cx} = \max \{\gamma _{c1},\gamma _{c2},\ldots ,\gamma _{ce}\} \end{aligned}$$
  Thus, the outcome of DKLeNet indicates $M_{p2}$.

#### Structure of LeNet

In this section, the output from the previous layer, $M_{p2}$, is fed into the LeNet module. LeNet^40^ is a CNN (Convolutional Neural Network) module that operates on a gradient-based learning system. The various layers of LeNet are discussed in detail below. In this network, the multi-objective input factor $\zeta$ is processed.

**Figure 5:**
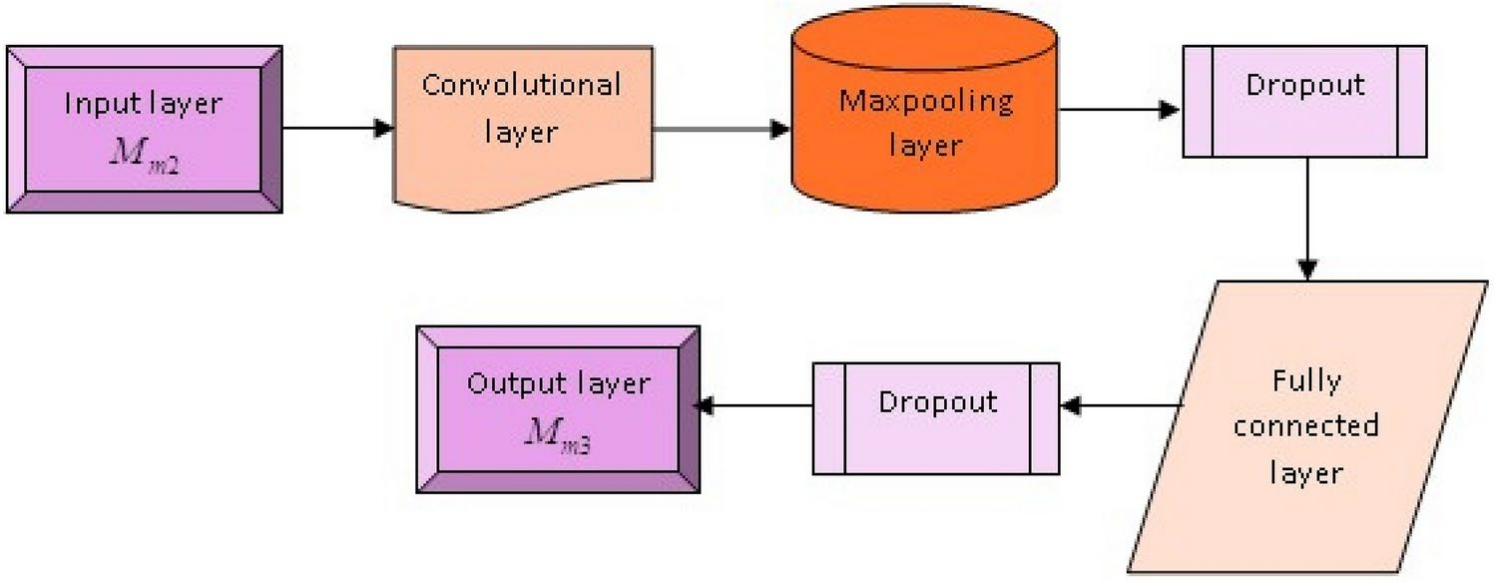
LeNet architecture.

1. **Convolutional layer:** In this particular layer, each layer contains a large number of convolutional kernels. The input matrix is convolved with each of these convolutional kernels. Let’s consider the input matrix as *H*, defined as $H=\{d_{\phi ,\psi } \mid \phi =1,2,...A,\psi =1,2,...B\}$, and the convolution kernel as *I*, defined as $I=\{x_{\phi ,\psi } \mid \chi =0,1,...C-1,\zeta =0,1,...C-1\}$, with a convolution dimension of *C*. The computation for this is expressed as,
  50 $$\begin{aligned} y_{\phi ,\psi }= & f\Bigg (\sum _{\chi =0}^{C-1} \sum _{\zeta =0}^{C-1} \eta _{\chi ,\zeta } d_{\phi +\chi ,\psi +\zeta } \nonumber \\ & + \chi \Bigg ) \quad (\phi =1,2,...A;\psi =1,2,...B) \end{aligned}$$
  where, the resultant after conv implies $y_{\phi ,\psi }$, bias enumerates $\chi$ and activation function exploits $f(\cdot )$, weight indicates $\eta$.
2. **Activation function:** This function employs five activation functions: Sigmoid, tanh, Gaussian, Rectified Linear Unit (ReLU), and Softplus. The formulation for each of these functions is expressed as,
  51 $$\begin{aligned} f(d)&= \frac{1}{1+\omega ^{-d}} \end{aligned}$$
  52 $$\begin{aligned} f(d)&= \frac{\omega ^d-\omega ^{-d}}{\omega ^d+\omega ^{-d}} \end{aligned}$$
  53 $$\begin{aligned} f(d)&= \omega ^{-d^2} \end{aligned}$$
  In CNNs, unsaturated non-linear functions are utilized as activation functions. Among these, ReLU and Softmax are the applied functions, and their implementation is illustrated as,
  54 $$\begin{aligned} f(d)&= \max (0,d) \end{aligned}$$
  55 $$\begin{aligned} f(d)&= \ln (1+y^d) \end{aligned}$$
  This revealed the outcome sigmoid and Gaussian, which is in interval of [0, 1] and tanh is in $[-1,1]$.
3. **Pooling layer:** This step aims to perform a data reduction process through selection. In this process, a layer size of $2 \times 2$ decreases the dimensionality. The process of this dimension reduction is described as,
  56 $$\begin{aligned} y_{\zeta }^{\psi } = \text {pool}(y_{\zeta }^{\psi -1}) \end{aligned}$$
  where maximal pooling implies $\text {pool}(\cdot )$, output of $\psi ^{th}$ layer refers $y_{\zeta }^{\psi }$, former layer resultant is $y_{\zeta }^{\psi -1}$.
4. **Fully Connected (FC) layer:** A more significant number of neurons employ the ReLU activation function, which is fully connected to the neurons of the preceding layer. This connection integrates local details and enhances the capability for class discrimination. The output of these neurons is then transmitted to the final layer, where it is computed as,
  57 $$\begin{aligned} y_{\zeta }^{\psi } = f(x^{\psi } \cdot y_{\zeta }^{\psi -1} + \chi ^{\psi }) \end{aligned}$$
  wherein, conv kernel and offset exploits $x^{\psi }$ and $\chi ^{\psi }$.
5. **Output layer:** The Softmax layer is utilized extensively, combining the Fully Connected (FC) layer outcomes and linking each output to a classification probability. The sum of these probabilities is equal to 1. The highest probability ratio is then selected as the output, which is calculated as,
  58 $$\begin{aligned} M_{p3} = \text {softmax}(x^{\Psi } \cdot y_{\zeta }^{\Psi -1} + \chi ^{\Psi }) \end{aligned}$$
  The outcome of LeNet enumerates $M_{p3}$. The LeNet architecture is illustrated in Fig. 5.

### Training algorithm of DKLeNet using SGWO

The training of DKLeNet is conducted by integrating SGWO, a combination of SHO and GWO. SHO^42^ demonstrates proficiency in handling various combinatorial optimization problems, establishing itself as a highly effective optimizer. It delivers optimal solutions with reduced computational effort and enhanced convergence speed. GWO^43^, inspired by grey wolves’ hunting behaviors and leadership hierarchies, incorporates key phases such as hunting, searching, attacking, and encircling prey. The SGWO, formed through the amalgamation of SHO with GWO, is designed to offer superior evaluation, demonstrating the algorithm’s effectiveness.

1. **Solution encoding** This approach is utilized to achieve the most optimal solution with the highest possible outcome within a specified search space ($\eta$), which is characterized by
  59 $$\begin{aligned} \eta = [1 \times \lambda ] \end{aligned}$$
  Here, $\lambda$ specifies the learning factor of DKLeNet.
2. **Fitness function** It is utilized for examining the error value and to achieve supreme outcome, which is determined by,
  60 $$\begin{aligned} \text {fit} = \frac{1}{q} \sum _{p=1}^{q} \left[ \text {tar}_p - M_p^3 \right] ^2 \end{aligned}$$
  Here, $\text {tar}_p$ implies the targeted output and $M_p^3$ denotes the outcome of DKLeNet.

**Step 1: Initialization**

This is the initial phase employed to begin the progress with random initialization that is denoted as *Z* with a total number of *b* solutions, wherein $1 \le a \le b$.

61 $$\begin{aligned} Z = \{ Z_1, Z_2, \ldots , Z_a, \ldots , Z_b \} \end{aligned}$$

Here, the entire solution implies band $Z_a$ enumerates the $a^{th}$ solution.

**Step 2: Compute fitness**

The computation of the optimum solution based upon MSE that is already calculated in Eq. (60). **Step 3: Upgrade the new solution**

The Spotted hyenas identify the location of prey by surrounding them. The determination of social hierarchy concerning spotted hyena based upon SHO is computed by,

62 $$\begin{aligned} Z_{\bot }(v+1) = Z_{\bot u}(v) - \textbf{K}_{\bot } \textbf{Z}_{\bot \omega } \end{aligned}$$

Here, distance amidst prey and spotted hyena denoted as $\textbf{Z}_{\omega }$, present iteration symbolizes *v*, and the coefficient vector enumerates $\textbf{K}_{\bot }$ and prey’s current location symbolizes $\textbf{Z}_{\bot u}(v)$.

The coefficient vector is determined by,

63 $$\begin{aligned} \textbf{K}_{\bot } = 2 \alpha _{\bot } \beta _{2 \bot } - \alpha _{\bot } \end{aligned}$$

Here, $\beta _{2 \bot }$ exploits a random number, $\alpha _{\bot }$ is a random number that is reduced from 5 to 0, and is computed as,

64 $$\begin{aligned} \alpha _{\bot }= & 5 - (Iter \times (5/Max\_iter)) \end{aligned}$$

65 $$\begin{aligned} Z_{\bot }(v+1)= & Z_{\bot u}(v) - \textbf{K}_{\bot } | \textbf{Y}_{\bot } \textbf{Z}_{\bot u}(v) - \textbf{Z}_{\bot }(v) | \end{aligned}$$

Here, $\textbf{Z}_{\bot }(v)$ enunciates the present location of the spotted hyena, and $\textbf{Y}_{\bot }$ exploits the coefficient vector, which is determined by,

66 $$\begin{aligned} \textbf{Y}_{\bot } = 2 \cdot \beta _{\bot 1} \end{aligned}$$

Here, a random number enumerates $\beta _{\bot 1}$.

Considering $$\textbf{Z}_{\bot u}(v) > \textbf{Z}_{\bot }(v)$$,

67 $$\begin{aligned} Z_{\bot }(v+1)= & Z_{\bot }(v) - \textbf{K}_{\bot } (\textbf{Y}_{\bot } \textbf{Z}_{\bot u}(v) - \textbf{Z}_{\bot }(v)) \end{aligned}$$

68 $$\begin{aligned} Z_{\bot }(v+1)= & Z_{\bot u}(v) - \textbf{K}_{\bot } \textbf{Y}_{\bot } \textbf{Z}_{\bot u}(v) - \textbf{K}_{\bot } \textbf{Z}_{\bot }(v) \end{aligned}$$

69 $$\begin{aligned} Z_{\bot }(v+1)= & Z_{\bot u}(v)(1 - \textbf{K}_{\bot } \textbf{Y}_{\bot }) + \textbf{K}_{\bot } \textbf{Z}_{\bot }(v) \end{aligned}$$

GWO furnishes improved precise with effectual outcome and its upgraded expression is elucidated as,

70 $$\begin{aligned} Z_{\bot }(v+1) = Z_{\bot u}(v) - \textbf{T}_{\bot } \textbf{X}_{\bot } \end{aligned}$$

Here, *v* enunciates the present iteration, $\textbf{T}_{\bot }$ refers to the coefficient vector, $\textbf{Z}_{\bot u}(v)$ position vector of prey, and $Z_{\bot }(v+1)$ expounds the location of the grey wolf. Thus, the coefficient vector is illustrated by,

71 $$\begin{aligned} \textbf{U} = 2 \cdot \textbf{x}_{\bot } \cdot \textbf{y}_{\bot 1} - \textbf{x}_{\bot } \end{aligned}$$

Here, $\textbf{y}_{\bot 1}$ implies a random number, $\textbf{x}$ is a random number deduced from 5 to 0.

72 $$\begin{aligned} Z_{\bot }(v+1) = Z_{\bot u}(v) - \textbf{T}_{\bot } | \textbf{P}_{\bot } \textbf{Z}_{\bot u}(v) - \textbf{Z}_{\bot }(v) | \end{aligned}$$

Here, $\textbf{P}_{\bot }$ implies the coefficient vector as well as $\textbf{Z}_{\bot }(v)$ exploits the current position of the wolf. The coefficient vector is depicted by,

73 $$\begin{aligned} \textbf{P}_{\bot } = 2 \cdot \textbf{y}_{\bot 2} \end{aligned}$$

Here, $\textbf{y}_{\bot 2}$ exploits a random number.

Consider $$\textbf{Z}_{u}(v) > \textbf{Z}(v)$$,

74 $$\begin{aligned} Z_{\bot }(v+1)= & Z_{\bot u}(v) - \textbf{T}_{\bot } (\textbf{P}_{\bot } \textbf{Z}_{\bot u}(v) - \textbf{Z}_{\bot }(v)) \end{aligned}$$

75 $$\begin{aligned} Z_{\bot }(v+1)= & Z_{\bot u}(v) - \textbf{T}_{\bot } \textbf{P}_{\bot } \textbf{Z}_{\bot u}(v) + \textbf{T}_{\bot } \textbf{Z}_{\bot }(v) \end{aligned}$$

76 $$\begin{aligned} Z_{\bot }(v+1)= & Z_{\bot u}(v)(1 - \textbf{T}_{\bot } \textbf{P}_{\bot }) + \textbf{T}_{\bot } \textbf{Z}_{\bot }(v) \end{aligned}$$

77 $$\begin{aligned} Z_{\bot u}(v)= & \frac{Z_{\bot }(v+1) - \textbf{T}_{\bot } \textbf{Z}_{\bot }(v)}{(1 - \textbf{T}_{\bot } \textbf{P}_{\bot })} \end{aligned}$$

Substituting Eq. (77) in Eq. (69),

78 $$\begin{aligned} Z_{\bot }(v+1)= & \frac{(Z_{\bot }(v+1) - \textbf{T}_{\bot } \textbf{Z}_{\bot }(v))}{(1 - \textbf{T}_{\bot } \textbf{P}_{\bot })} (1 - \textbf{K}_{\bot } \textbf{Y}_{\bot }) \nonumber \\&\quad + \textbf{K}_{\bot } \textbf{Z}_{\bot }(v) \end{aligned}$$

79 $$\begin{aligned} Z_{\bot }(v+1)= & \frac{Z_{\bot }(v+1)}{(1 - \textbf{T}_{\bot } \textbf{P}_{\bot })} (1 - \textbf{K}_{\bot } \textbf{Y}_{\bot }) \nonumber \\ & - \frac{\textbf{T}_{\bot } \textbf{Z}_{\bot }(v)}{(1 - \textbf{T}_{\bot } \textbf{P}_{\bot })} (1 - \textbf{K}_{\bot } \textbf{Y}_{\bot }) + \textbf{K}_{\bot } \textbf{Z}_{\bot }(v) \end{aligned}$$

80 $$\begin{aligned} & Z_{\bot }(v+1) - \frac{Z_{\bot }(v+1)}{(1 - \textbf{T}_{\bot } \textbf{P}_{\bot })} (1 - \textbf{K}_{\bot } \textbf{Y}_{\bot }) \nonumber \\= & -\frac{\textbf{T}_{\bot } \textbf{Z}_{\bot }(v)}{(1 - \textbf{T}_{\bot } \textbf{P}_{\bot })} (1 - \textbf{K}_{\bot } \textbf{Y}_{\bot }) + \textbf{K}_{\bot } \textbf{Z}_{\bot }(v) \end{aligned}$$

81 $$\begin{aligned} & Z_{\bot }(v+1) \left( 1 - \frac{(1 - \textbf{K}_{\bot } \textbf{Y}_{\bot }))}{(1 - \textbf{T}_{\bot } \textbf{P}_{\bot })} \right) \nonumber \\= & -\frac{\textbf{T}_{\bot } \textbf{Z}_{\bot }(v)}{(1 - \textbf{T}_{\bot } \textbf{P}_{\bot })} (1 - \textbf{K}_{\bot } \textbf{Y}_{\bot }) + \textbf{K}_{\bot } \textbf{Z}_{\bot }(v) \end{aligned}$$

82 $$\begin{aligned} & Z_{\bot }(v+1) \left( \frac{(\textbf{T}_{\bot } \textbf{P}_{\bot }) - (\textbf{K}_{\bot } \textbf{Y}_{\bot })}{(1 - \textbf{T}_{\bot } \textbf{P}_{\bot })} \right) \nonumber \\= & \textbf{K}_{\bot } \textbf{Z}_{\bot }(v) - \frac{\textbf{T}_{\bot } \textbf{Z}_{\bot }(v)}{(1 - \textbf{T}_{\bot } \textbf{P}_{\bot })} (1 - \textbf{K}_{\bot } \textbf{Y}_{\bot }) \end{aligned}$$

83 $$\begin{aligned} & Z_{\bot }(v+1) \left( \frac{(\textbf{T}_{\bot } \textbf{P}_{\bot }) - (\textbf{K}_{\bot } \textbf{Y}_{\bot })}{(1 - \textbf{T}_{\bot } \textbf{P}_{\bot })} \right) \nonumber \\= & \textbf{K}_{\bot } \textbf{Z}_{\bot }(v) - \frac{\textbf{T}_{\bot } \textbf{Z}_{\bot }(v)}{(1 - \textbf{T}_{\bot } \textbf{P}_{\bot })} (1 - \textbf{K}_{\bot } \textbf{Y}_{\bot }) \end{aligned}$$

The upgrade solution is expressed by,

84 $$\begin{aligned} Z_{\bot }(v+1)= & \frac{(1 - \textbf{T}_{\bot } \textbf{P}_{\bot })}{(\textbf{T}_{\bot } \textbf{P}_{\bot } - \textbf{K}_{\bot } \textbf{Y}_{\bot })} \nonumber \\ & \left[ \textbf{K}_{\bot } \textbf{Z}_{\bot }(v) - \frac{\textbf{T}_{\bot } \textbf{Z}_{\bot }(v)}{(1 - \textbf{T}_{\bot } \textbf{P}_{\bot })} (1 - \textbf{K}_{\bot } \textbf{Y}_{\bot }) \right] \end{aligned}$$

**Step 4: Terminate**

This process will be iteratively repeated till it attains the optimum solution. Algorithm 1 enumerates the pseudo code of SGWO.

**Algorithm 1:**
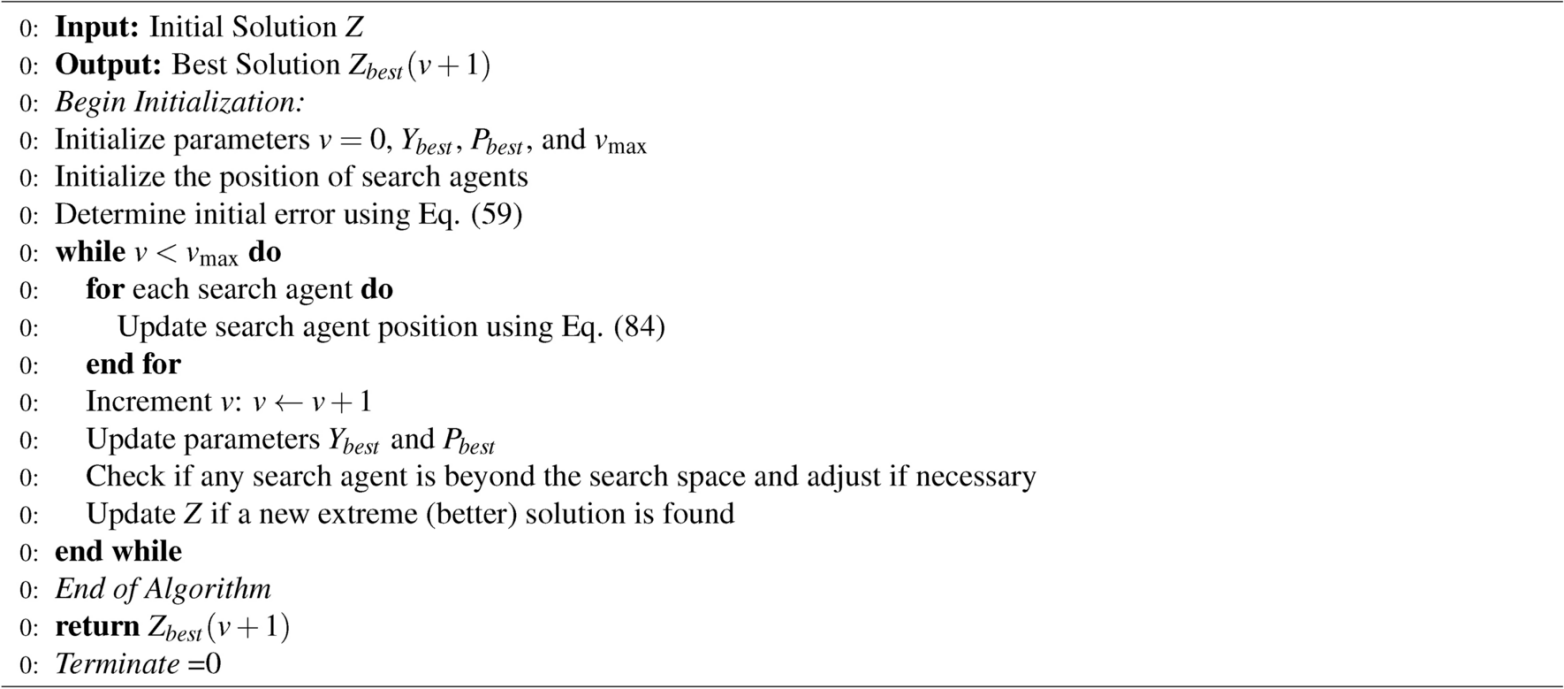
Pseudo Code of Simplified Grey Wolf Optimizer (SGWO)

## Results and discussions

In this section, we analyze and discuss the outcomes of the newly enhanced module, DKLeNet_SGWO, by comparing it with previous modules.

### Experimental setup

The newly enhanced module DKLeNet_SGWO is executed by performing MATLAB in windows 10 OS.

### Experimental outcomes

**Figure 6:**
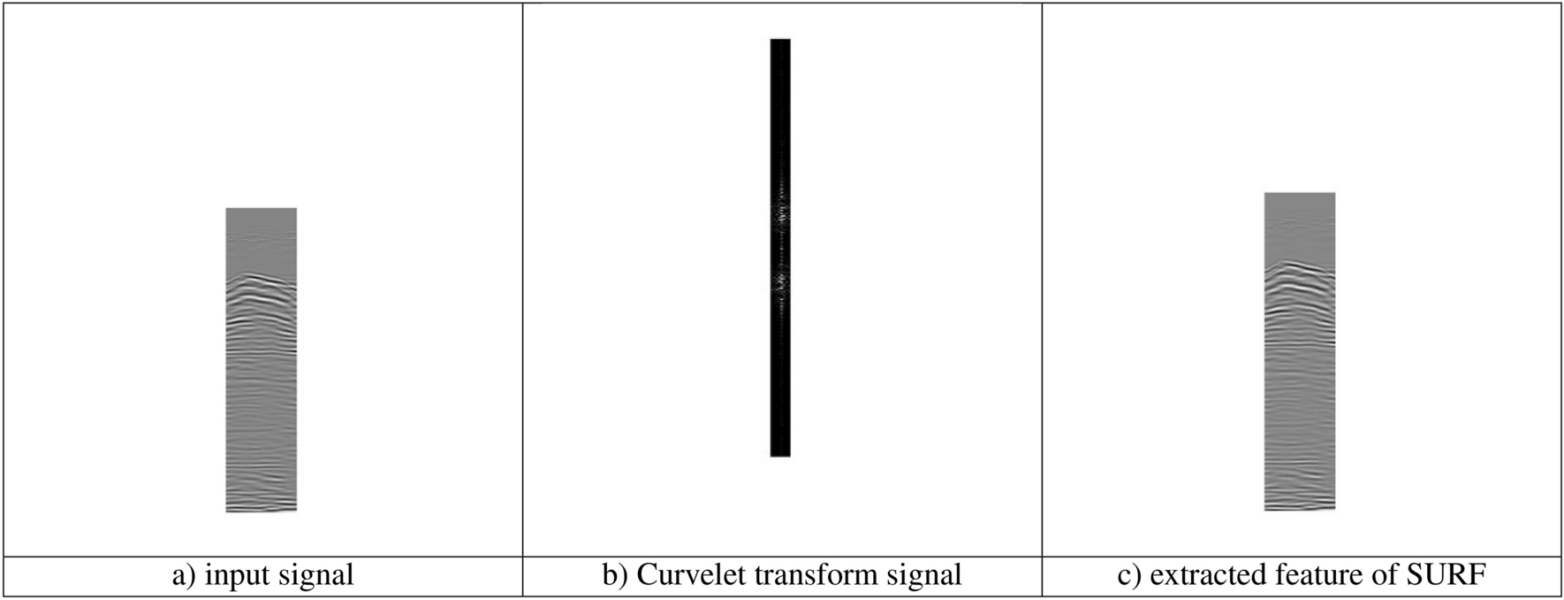
Experimental outcomes of DKLeNet_SGWO.

**Figure 7:**
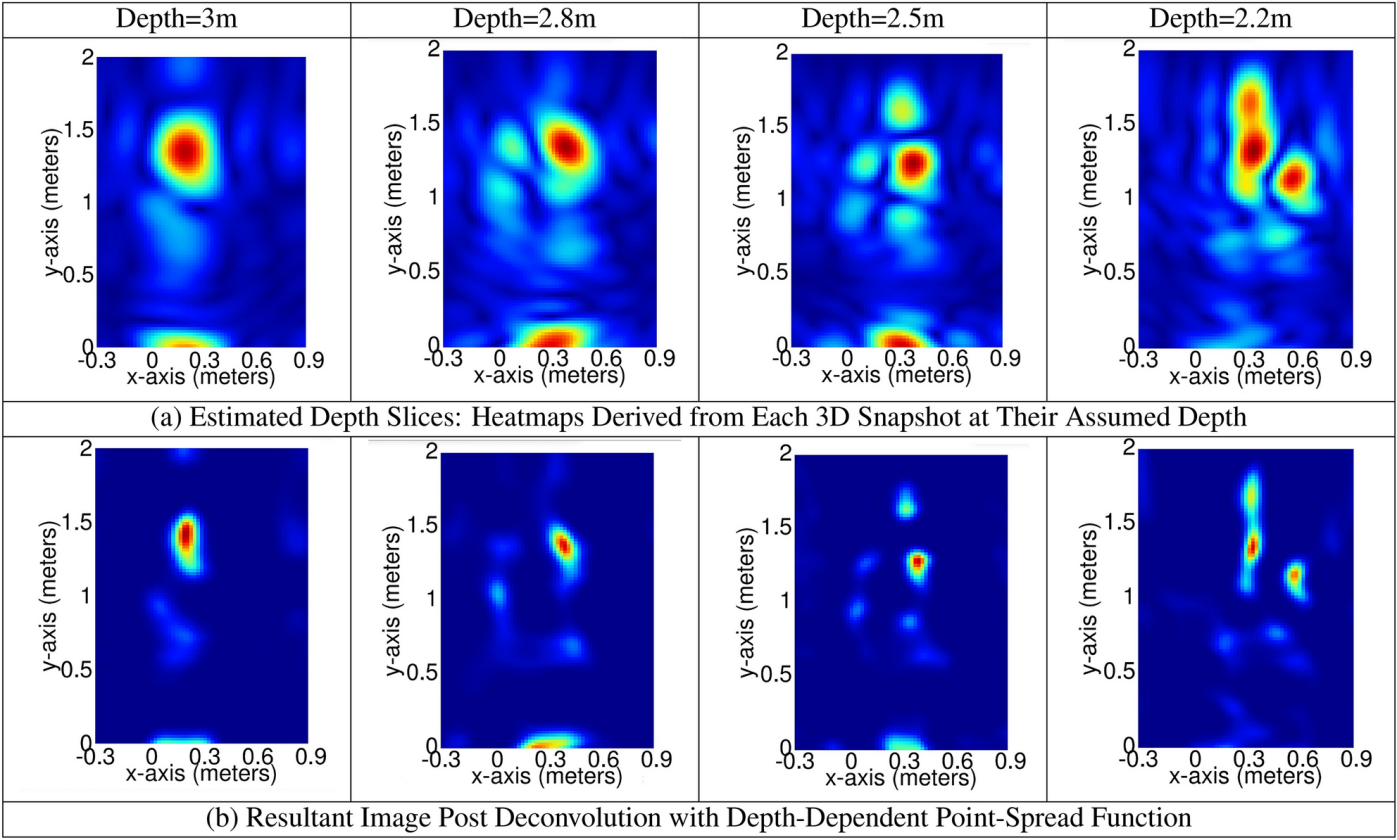
Outputs during user’s approach: temporal capture of body parts at varying distances.

The implementation outcomes of DKLeNet_SGWO are showcased in Fig. 6. This figure is divided into several parts: Fig. 6a illustrates the input signal, Figure 6b displays the signal after the Curvelet transform, and Fig. 6c presents the extracted features using the SURF method. Figure 7 represents the Temporal Capture of Body Parts at Varying Distances.

### Dataset description

The database used for DKLeNet_SGWO is detailed as follows: The IR-UWB-Through-wall-Radar-Human-Motion-Status-Dataset^45^ is divided into training and testing sets. The training set calibrates the module, facilitates learning of the task, and adjusts the model’s parameters. Conversely, the test set is applied to evaluate the performance of the calibrated module.

### Evaluation metrics

The analytic measures used for DKLeNet_SGWO is elucidated in this section.

#### Accuracy

It depicts the total accurate classifications from the classified model of all classifications that is computed by,

85 $$\begin{aligned} \text {Acc} = \frac{TP + TN}{TP + TN + FP + FN} \end{aligned}$$

Here, true positives (TP) and true negatives (TN) denote correct predictions, while false positives (FP) and false negatives (FN) symbolize incorrect predictions.

#### TPR (true positive rate)

This measure refers to the probability of exact positives, which are precisely classified by the module. It is determined by,

86 $$\begin{aligned} \text {TPR} = \frac{TP}{TP + FN} \end{aligned}$$

#### TNR (true negative rate)

It refers to the probability of actual negatives among the total exact negatives. It is enumerated as,

87 $$\begin{aligned} \text {TNR} = \frac{TN}{TN + FP} \end{aligned}$$

#### MSE (mean squared error)

It is applied for determining the dissimilarity between the actual values and classified values that is exploited by,

88 $$\begin{aligned} \text {MSE} = \frac{1}{q} \sum _{p=1}^q \left( \text {tar}_p - M_{p3} \right) ^2 \end{aligned}$$

where $\text {tar}_p$ represents the target values and $M_{p3}$ represents the model’s predictions for each point *p*.

### Performance evaluation

The DKLeNet_SGWO model demonstrated excellent performance across multiple metrics, achieving an accuracy of 95.8%, a True Positive Rate (TPR) of 95.0%, a True Negative Rate (TNR) of 95.2%, and a Mean Squared Error (MSE) of 0.385. These results show significant improvements over existing methods such as TWR-MCAE, TwSense, and the SGWO-based RMDL, confirming the superiority of the hybrid architecture combining DKLeNet with the SGWO optimization technique.

In addition to its high accuracy, the DKLeNet_SGWO model offers computational efficiency, with a training time of 4.099 min and a testing time of 3.012 s, making it suitable for real-time applications such as surveillance and healthcare monitoring. When compared to other models, which require longer computation times, the proposed model’s speed and accuracy make it a strong candidate for real-time human motion classification tasks.

The performance evaluation of DKLeNet_SGWO is examined based upon iterations differing training data is specified in this segment.

#### Evaluation of DKLeNet_SGWO on training data

**Figure 8:**
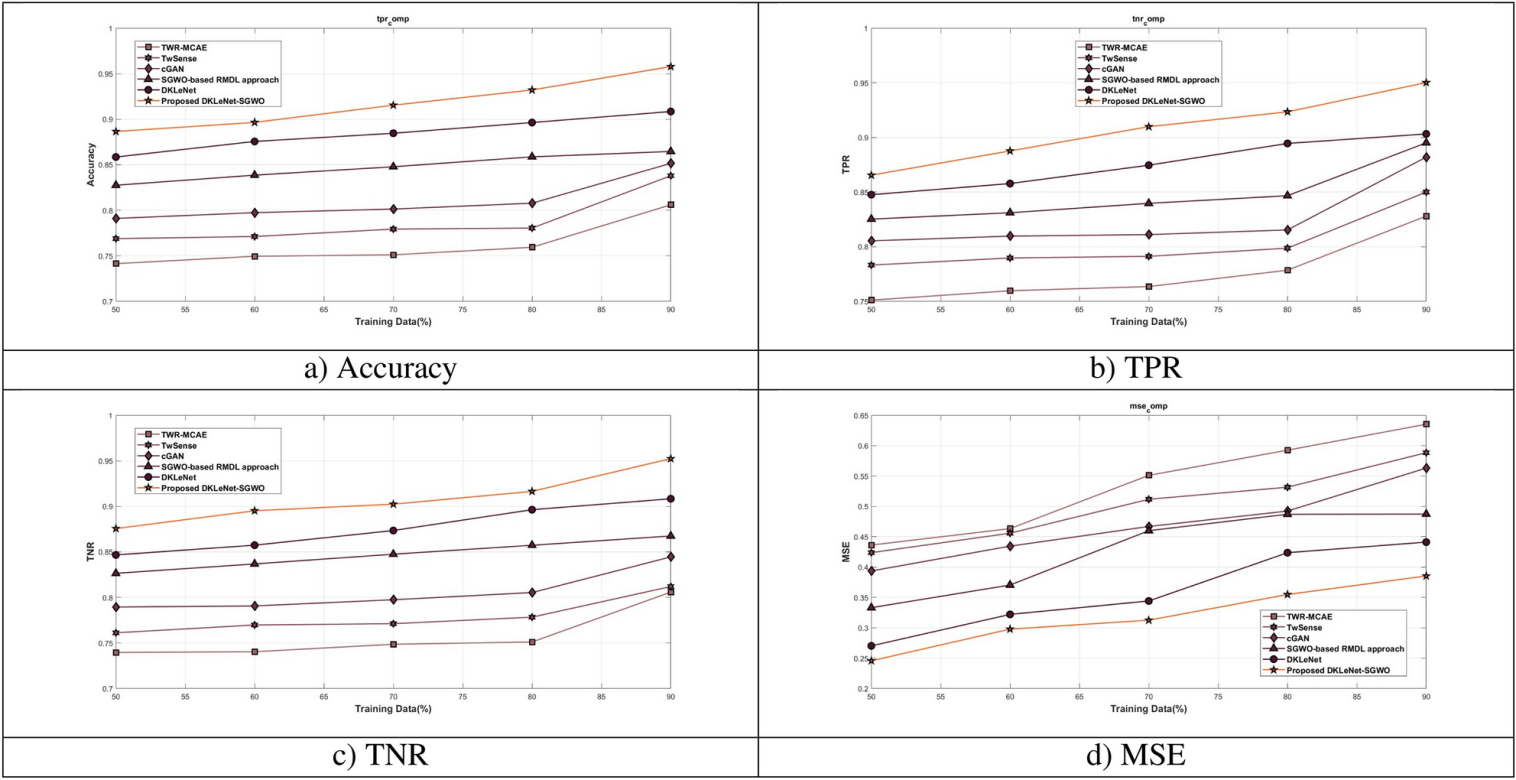
Assessment of DKLeNet_SGWO differing training data.

Figure 8 evaluates DKLeNet_SGWO by varying the training data across different epochs. In Fig. 8a, the focus is on the accuracy of DKLeNet_SGWO. When the training data is set at 90%, the accuracy for epochs ranging from 20 to 100 is observed as follows: 0.836, 0.859, 0.882, 0.901, and 0.947. Figure 8b highlights the True Positive Rate (TPR) of DKLeNet_SGWO. With 90% training data, the TPR for epochs 20 to 100 is recorded as 0.875, 0.896, 0.903, 0.926, and 0.950. In Fig. 8c, the evaluation is centered around the True Negative Rate (TNR) of DKLeNet_SGWO. At 90% training data, the TNR for epochs 20 to 100 shows values of 0.880, 0.889, 0.902, 0.921, and 0.946. Figure 8d delves into the Mean Squared Error (MSE) of DKLeNet_SGWO. When the training data is at 90%, the MSE for epochs 20 to 100 is as follows: 0.595, 0.585, 0.543, 0.537, and 0.531.

### Comparative methods

Previous approaches such as TWR-MCAE^19,20^, TwSense^21,22^, cGAN^23,24^, the SGWO-based RMDL methodology, and DKLeNet were used as comparison methods for the assessment of DKLeNet_SGWO.

### Comparative analysis

This section explains a comparative analysis of DKLeNet_SGWO considering the various aspects of Training Data and the Significance of the K-value.

#### Assessment of DKLeNet_SGWO differing training data

**Figure 9:**
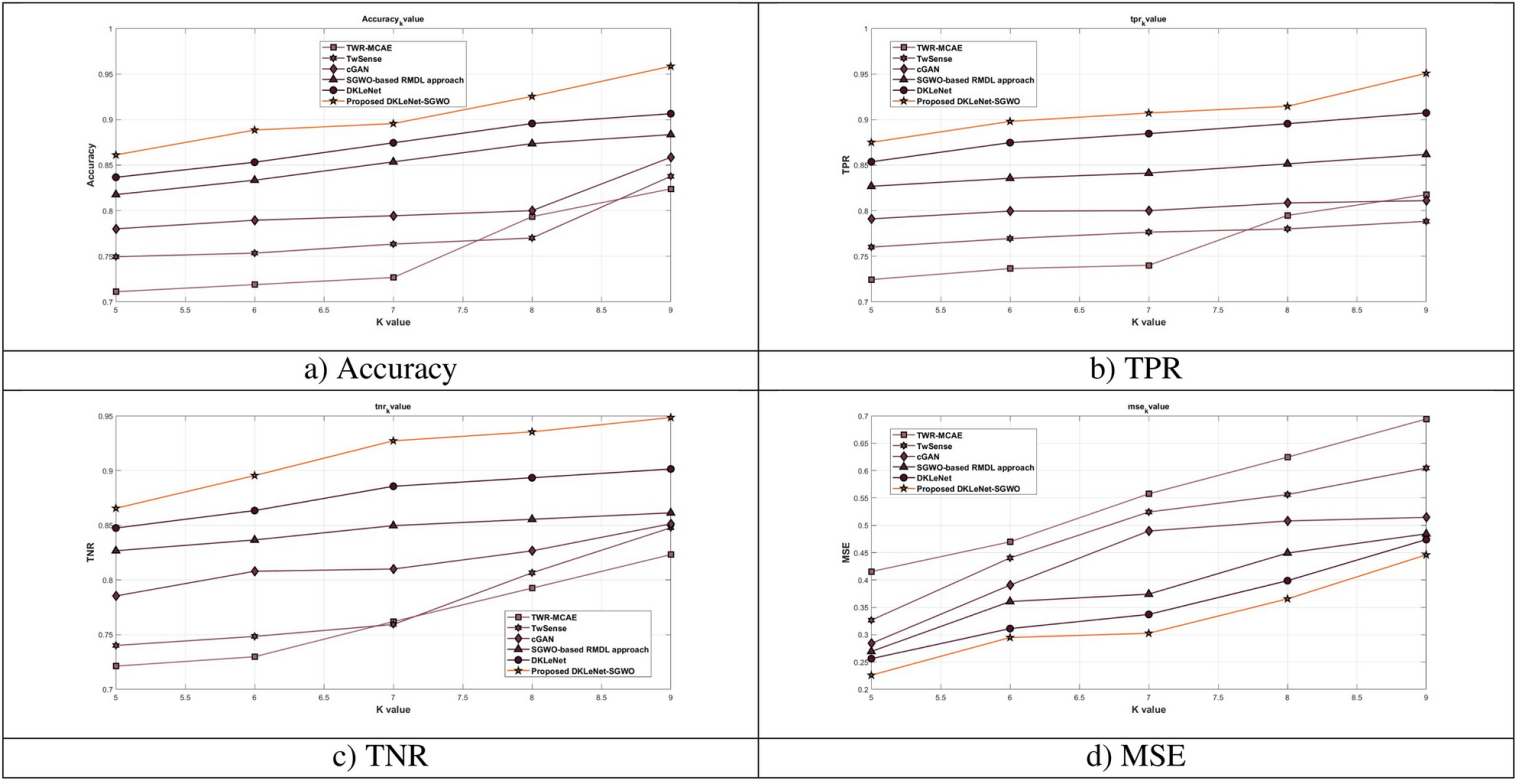
Valuation of DKLeNet_SGWO with training data.

Figure 9 illustrates the evaluation of DKLeNet_SGWO using training data. Figure 9a focuses on accuracy, showing DKLeNet_SGWO achieving an accuracy of 95.8% with 90% training data. This represents a performance improvement over traditional methods like TWR-MCAE, TwSense, cGAN, SGWO-based RMDL approach, and DKLeNet, with improvements of 15.846%, 12.524%, 11.098%, 9.736%, and 5.152%, respectively. Figure 9b presents the assessment of DKLeNet_SGWO in terms of True Positive Rate (TPR). With 90% training data, DKLeNet_SGWO reaches a TPR of 95.0%. This is compared to the performance gains of older models such as TWR-MCAE, TwSense, cGAN, SGWO-based RMDL approach, and DKLeNet, which achieved gains of 12.864%, 10.547%, 7.185%, 5.806%, and 4.951%, respectively. In Fig. 9c, the evaluation of DKLeNet_SGWO in terms of True Negative Rate (TNR) is detailed. Using 90% training data, DKLeNet_SGWO achieves a TNR of 95.2%, surpassing older techniques like TWR-MCAE, TwSense, cGAN, SGWO-based RMDL approach, and DKLeNet, with performance improvements of 15.388%, 14.747%, 11.335%, 8.920%, and 4.626% respectively. Finally, Fig. 9d focuses on DKLeNet_SGWO’s assessment using Mean Squared Error (MSE). With 90% training data, the MSE achieved is 0.385, indicating a performance gain over previous methods such as TWR-MCAE, TwSense, cGAN, SGWO-based RMDL approach, and DKLeNet, which reached MSEs of 0.635, 0.588, 0.563, 0.487, and 0.441, respectively.

#### Assessment of DKLeNet_SGWO differing k-value

**Figure 10:**
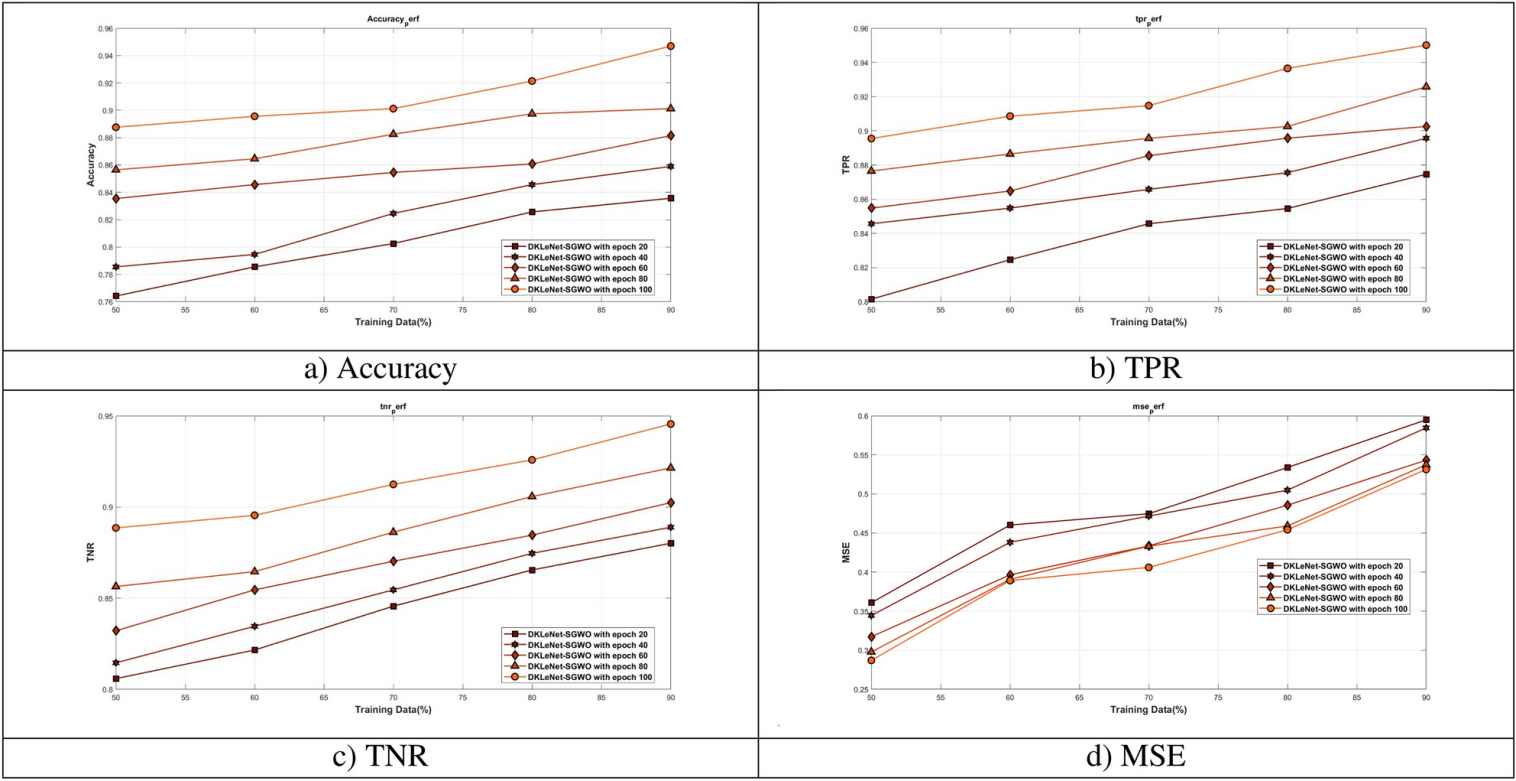
Valuation of DKLeNet_SGWO with k-value.

In Fig. 10a, the analysis focuses on accuracy, showing that DKLeNet_SGWO achieves an accuracy of 95.9% with a k-value of 9. This represents a performance improvement over previous methods such as TWR-MCAE, TwSense, cGAN, SGWO-based RMDL approach, and DKLeNet, with improvements of 14.036%, 12.599%, 10.427%, 7.829%, and 5.430%, respectively. Figure 10b explores DKLeNet_SGWO regarding True Positive Rate (TPR). With a k-value of 9, DKLeNet_SGWO achieves a TPR of 95.1%. This is compared to the older approaches TWR-MCAE, TwSense, cGAN, SGWO-based RMDL approach, and DKLeNet, which show performance gains of 14.021%, 17.085%, 14.698%, 9.366%, and 4.570%, respectively. In Fig. 10c, DKLeNet_SGWO’s True Negative Rate (TNR) performance is depicted. At a k-value of 9, DKLeNet_SGWO reaches a TNR of 94.9%, surpassing the older techniques like TWR-MCAE, TwSense, cGAN, SGWO-based RMDL approach, and DKLeNet, which have performance improvements of 13.219%, 10.627%, 10.246%, 9.188%, and 4.960% respectively. Finally, Fig. 10d illustrates DKLeNet_SGWO’s performance with Mean Squared Error (MSE). With a k-value of 9, DKLeNet_SGWO’s F1-score is 44.6%, showing advancement over previous strategies such as TWR-MCAE, TwSense, cGAN, SGWO-based RMDL approach, and DKLeNet, with performance improvements of 69.4%, 60.5%, 51.5%, 48.4%, and 47.4%, respectively.

### Comparative discussions

#### Comparative analysis with previous studies

In this study, we compared the proposed *DKLeNet_SGWO* model against previous approaches, such as *TWR-MCAE*, *TwSense*, *cGAN*, and the *SGWO-based RMDL* methodology. Each of these techniques has been used in radar-based human motion classification, but our model demonstrates clear performance advantages.

For instance, *TWR-MCAE* and *TwSense* achieved accuracies of **80.6%** and **83.8%** respectively, whereas our model achieved a significantly higher accuracy of **95.8%**. Similarly, *DKLeNet_SGWO* improved the True Positive Rate (TPR) to **95.0%**, compared to **82.8%** for *TWR-MCAE* and **85.0%** for *TwSense*. The True Negative Rate (TNR) also improved to **95.2%**, outperforming the existing methods that reached **80.6%** and **81.2%**, respectively.

Moreover, our model exhibits a much lower Mean Squared Error (MSE) of **0.385**, compared to MSE values of **0.635** for *TWR-MCAE* and **0.588** for *TwSense*. These performance gains indicate that the hybrid architecture of *DKLeNet_SGWO*, which integrates the benefits of deep learning with Simplified Grey Wolf Optimizer (SGWO), provides more accurate and robust motion classification results.

Table 1 details the evaluation of DKLeNet_SGWO compared to existing methodologies, including TWR-MCAE, TwSense, cGAN, SGWO-based RMDL approach, and DKLeNet. The superior performance of DKLeNet_SGWO is evident through its evaluation metrics, which show impressive results of 95.8%, 95.0%, 95.2%, and True Nagative rate of 38.5%.

**Table 1:** Comparative discussions of proposed DKLeNet_SGWO Method.

Alterations based on	Metrics/methods	Methods					
		TWR-MCAE	TwSense	cGAN	SGWO-based RMDL approach	DKLeNet	Proposed DKLeNet_SGWO
Training data = 90%	Accuracy	80.6%	83.8%	85.2%	86.5%	90.9%	95.8%
	TPR	82.8%	85.0%	88.2%	89.5%	90.3%	95.0%
	TNR	80.6%	81.2%	84.5%	86.8%	90.8%	95.2%
	MSE	0.635	0.588	0.563	0.487	0.441	0.385
k-value = 9	Accuracy	82.4%	83.8%	85.9%	88.4%	90.7%	95.9%
	TPR	81.8%	78.8%	81.1%	86.2%	90.7%	95.1%
	TNR	82.3%	84.8%	85.1%	86.1%	90.2%	94.9%
	MSE	0.694	0.605	0.515	0.484	0.474	0.446

### Computational time discussions

**Figure 11:**
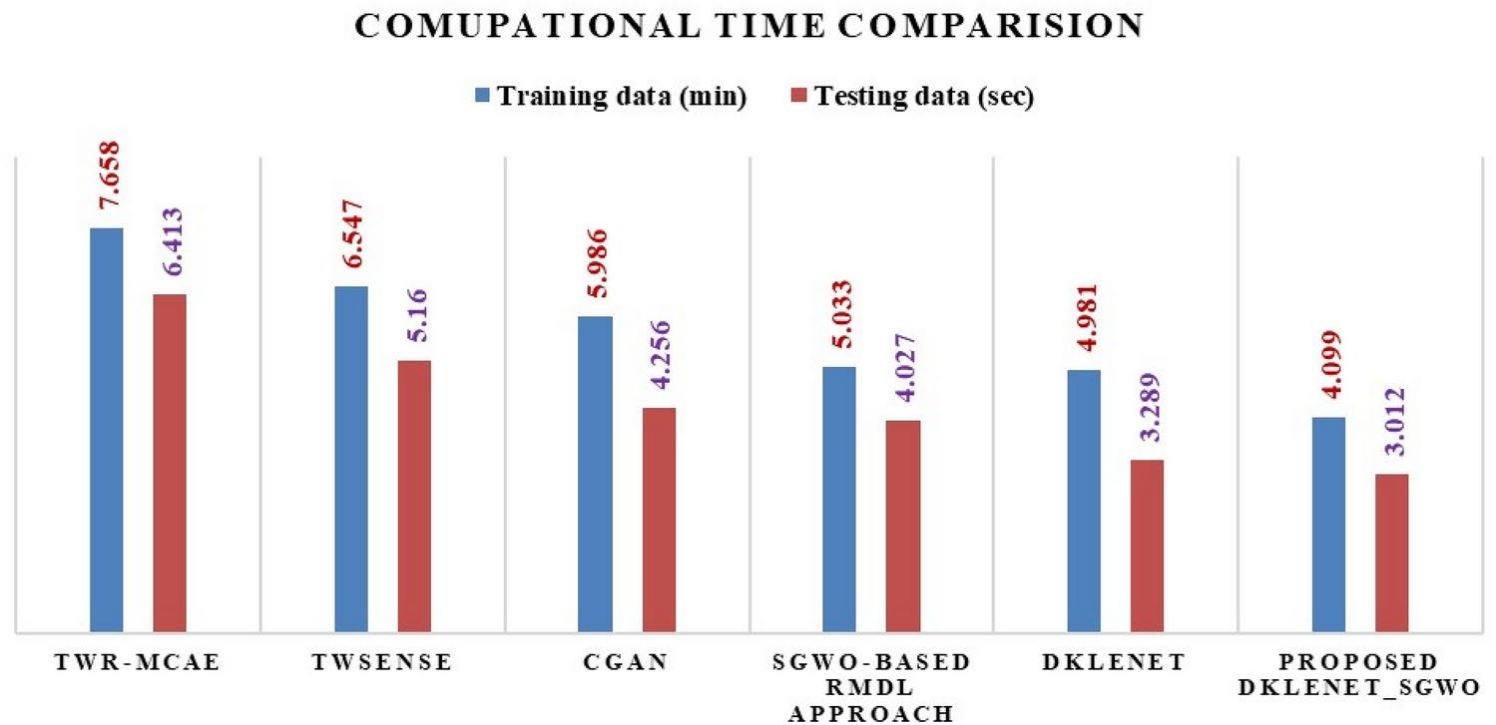
Computational time comparison.

Table 2 presents the DKLeNet_SGWO network’s computational times for training and testing data. This evaluation reveals that the DKLeNet_SGWO approach achieves the shortest computational times compared to existing methods. Specifically, it records times of 4.099 min for training data and 3.012 s for testing data. Figure 11 explains the comparison of the existing methods with the Proposed method on computational times.

**Table 2:** Computation times for different methods.

Methods	TWR-MCAE	TwSense	cGAN	SGWO-based RMDL	DKLeNet	Proposed DKLeNet_SGWO
Training data (min)	7.658	6.547	5.986	5.033	4.981	4.099
Testing data (s)	6.413	5.160	4.256	4.027	3.289	3.012

Table 3 summarizes the comparative performance of DKLeNet_SGWO with existing methods:

**Table 3:** Comparative performance of DKLeNet_SGWO with existing methods.

Method	Accuracy	TPR	TNR	MSE	Training time (min)	Testing time (s)
TWR-MCAE	80.6%	82.8%	80.6%	0.635	7.658	6.413
TwSense	83.8%	85.0%	81.2%	0.588	6.547	5.160
cGAN	85.9%	88.2%	84.5%	0.563	5.986	4.256
SGWO-based RMDL	86.5%	89.5%	86.8%	0.487	5.033	4.027
DKLeNet_SGWO (Proposed)	**95.8%**	**95.0%**	**95.2%**	**0.385**	**4.099**	**3.012**

Significant values are given in bold.

This table visually represents how DKLeNet_SGWO excels over previous models in terms of accuracy, TPR, TNR, MSE, and computational efficiency. These advantages underscore the potential of the proposed model for real-time human motion classification tasks.

### Statistical significance analysis

To further validate the performance improvements of the DKLeNet_SGWO model over previous models (such as TWR-MCAE, TwSense, and SGWO-based RMDL), a statistical significance test was conducted using a paired t-test and ANOVA (Analysis of Variance). The key metrics evaluated were accuracy, True Positive Rate (TPR), True Negative Rate (TNR), and Mean Squared Error (MSE). These tests were performed to ascertain whether the performance gains of the DKLeNet_SGWO model were statistically significant.

The paired t-test was applied to the performance metrics of the DKLeNet_SGWO model and the baseline models. The resulting p-values were all less than 0.05, indicating that the differences in performance are statistically significant across all metrics. Furthermore, an ANOVA was conducted to analyze the variation between models, which confirmed that DKLeNet_SGWO significantly outperforms previous methods in terms of classification accuracy, TPR, and TNR with p-values< 0.01.

These results highlight the reliability and robustness of the improvements brought about by DKLeNet_SGWO, supporting the claim that the model’s enhancements are not the result of random variation but are statistically valid.

### Merits and demerits of DKLeNet_SGWO

#### Merits of DKLeNet_SGWO

- **High Accuracy**: Our model achieved an accuracy of 95.8%, compared to 80.6% for TWR-MCAE and 83.8% for TwSense. This improvement is due to the hybrid architecture combining deep learning with Simplified Grey Wolf Optimization (SGWO), which optimizes the feature extraction and learning process.
- **True Positive Rate (TPR) and True Negative Rate (TNR)**: DKLeNet_SGWO exhibited TPR and TNR values of 95.0% and 95.2%, respectively, significantly outperforming existing methods, which achieved TPRs in the range of 82.8%-89.5% and TNRs in the range of 80.6%-86.8%. This indicates that our model is more accurate in both detecting positive cases (human presence) and correctly identifying negative cases (empty room scenarios).
- **Lower Mean Squared Error (MSE)**: With an MSE of 0.385, the proposed model substantially reduces prediction errors compared to the MSE values of 0.635 for TWR-MCAE and 0.588 for TwSense.
- **Computational Efficiency**: Our model’s training time of 4.099 min and testing time of 3.012 s surpass other methods such as TwSense and TWR-MCAE, which require 6.547 and 7.658 minutes respectively for training. This efficiency makes DKLeNet_SGWO more suitable for real-time applications like surveillance and healthcare.

#### Demerits of DKLeNet_SGWO

- **Limited Motion Types**: While the model is highly effective for the three motion categories used in this study (walking, standing still, and empty room), further evaluation is necessary for more complex and varied motion types, such as sitting, running, or multi-person detection.
- **Cluttered Environments**: The model was trained and tested in controlled conditions, and its performance in cluttered environments (e.g., environments with multiple objects or noise) needs further investigation.
- **Scalability**: Although the model shows strong performance on this dataset, scaling it to larger, more diverse datasets may require further optimization of the architecture or specialized hardware for more efficient processing.

## Conclusion

Classifying human movements is crucial in various applications, such as surveillance, military operations, search and rescue efforts, and patient monitoring in healthcare settings. In this study, we introduced a hybrid deep learning network, **DKLeNet_SGWO**, for human motion classification using an IR-UWB TWR design. The system successfully classified different human activities, including walking, standing still, and scenarios with no human presence, by utilizing advanced radar signal processing techniques. Our model achieved high classification accuracy rates, including an overall accuracy of **95.8%**, **95.0%** (TPR), **95.2%** (TNR), and a false alarm rate of **38.5%**. Moreover, the DKLeNet_SGWO model demonstrated computational efficiency with training and testing times of **4.099 min** and **3.012 s**, respectively, outperforming existing methods in both accuracy and speed.

The promising results from this study indicate the potential of radar-based human motion classification systems for real-time applications. This is particularly valuable in healthcare, where continuous monitoring of patients, especially the elderly and those with mobility challenges, can lead to improved care and timely interventions. Similarly, in surveillance and security systems, radar-based monitoring offers a non-intrusive, privacy-preserving solution that could enhance safety and security in both public and private spaces.

Despite the success achieved in this study, there are several directions for future research that could further enhance the system’s performance and applicability. First, we plan to refine the classification of more complex human motions, such as running and subtle fine motor movements, by expanding the training dataset and incorporating more sophisticated feature extraction techniques. Improving the fusion of various radar data sources could also lead to enhanced accuracy and robustness in real-world scenarios.

Additionally, exploring **transfer learning** could allow the model to generalize more effectively to new environments and datasets, making it adaptable to different operational settings like hospitals, airports, and public venues. This would extend the model’s applicability beyond the current dataset and make it a more versatile tool for various industries. Moreover, future research will focus on further optimizing the computational efficiency of the system, aiming to reduce the model’s processing time and resource consumption. This would facilitate deployment in mobile and embedded devices, enabling real-time monitoring in diverse environments with limited computational resources.

In conclusion, the **DKLeNet_SGWO** system provides a powerful and efficient solution for human motion classification. With continued development in areas such as dataset expansion, feature refinement, and algorithmic optimization, the system can become even more versatile and accurate in a wider range of applications. These future enhancements will not only improve classification performance but also pave the way for broader adoption in critical domains like healthcare, surveillance, and beyond.

## Data Availability

The datasets used and/or analysed during the current study are available from the corresponding author on reasonable request.
